# Genomic overview of INA-induced NPR1 targeting and transcriptional cascades in Arabidopsis

**DOI:** 10.1093/nar/gkae019

**Published:** 2024-01-23

**Authors:** Se-Hun Yun, Irfan Ullah Khan, Bosl Noh, Yoo-Sun Noh

**Affiliations:** School of Biological Sciences, Seoul National University, Seoul 08826, Korea; Research Center for Plant Plasticity, Seoul National University, Seoul 08826, Korea; School of Biological Sciences, Seoul National University, Seoul 08826, Korea; Research Center for Plant Plasticity, Seoul National University, Seoul 08826, Korea; Research Institute of Basic Sciences, Seoul National University, Seoul 08826, Korea; School of Biological Sciences, Seoul National University, Seoul 08826, Korea; Research Center for Plant Plasticity, Seoul National University, Seoul 08826, Korea

## Abstract

The phytohormone salicylic acid (SA) triggers transcriptional reprogramming that leads to SA-induced immunity in plants. NPR1 is an SA receptor and master transcriptional regulator in SA-triggered transcriptional reprogramming. Despite the indispensable role of NPR1, genome-wide direct targets of NPR1 specific to SA signaling have not been identified. Here, we report INA (functional SA analog)-specific genome-wide targets of Arabidopsis NPR1 in plants expressing GFP-fused NPR1 under its native promoter. Analyses of *NPR1*-dependently expressed direct NPR1 targets revealed that NPR1 primarily activates genes encoding transcription factors upon INA treatment, triggering transcriptional cascades required for INA-induced transcriptional reprogramming and immunity. We identified genome-wide targets of a histone acetyltransferase, HAC1, including hundreds of co-targets shared with NPR1, and showed that *NPR1* and *HAC1* regulate INA-induced histone acetylation and expression of a subset of the co-targets. Genomic NPR1 targeting was principally mediated by TGACG-motif binding protein (TGA) transcription factors. Furthermore, a group of NPR1 targets mostly encoding transcriptional regulators was already bound to NPR1 in the basal state and showed more rapid and robust induction than other NPR1 targets upon SA signaling. Thus, our study unveils genome-wide NPR1 targeting, its role in transcriptional reprogramming, and the cooperativity between NPR1, HAC1, and TGAs in INA-induced immunity.

## Graphical Abstract

**Graphical Abstract:**
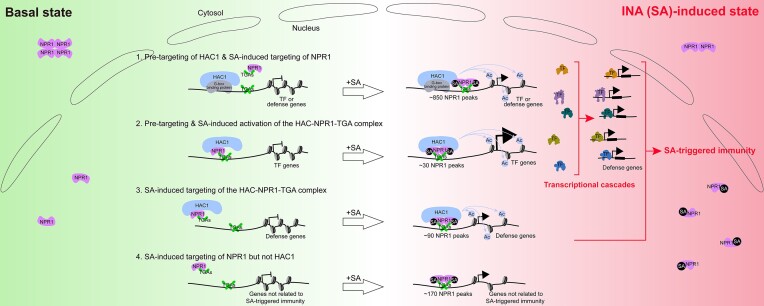

## Introduction

Throughout their lifetime, plants are threatened by pathogens. Unlike animals, plants do not differentiate specialized immune cells or organs, but instead transition their cell identity from a growth-optimized to an immunity-equipped state partially through genome-wide transcriptional reprogramming. Salicylic acid (SA) is a key phytohormone that induces disease resistance against biotrophic and hemi-biotrophic pathogens (1). Upon pathogen attack, plant SA levels increase, which induces genome-wide transcriptional reprogramming to elicit immune responses (2–4).

SA-triggered transcriptional reprogramming is dependent on NONEXPRESSOR OF PATHOGENESIS-RELATED GENES1 (NPR1). Thus, *npr1* mutants fail to develop disease resistance or express pathogenesis-related (*PR*) genes after SA treatment or pathogen challenge (5–8). Furthermore, NPR1 is an SA receptor (9,10) and undergoes a conformational change that enables its transcriptional co-activator function upon SA binding (9,11). In a microarray-based study, an *npr1* mutation affected the expression of 99% of genes that are induced by benzothiadiazole *S*-methylester (BTH; a functional SA analog) (4), and an RNA sequencing (RNA-seq)-based study showed that the expression of 71% of 2,6-dichloroisonicotinic acid (INA; a functional SA analog)-inducible genes is affected by an *npr1* mutation (3), all supporting an essential and central role of NPR1 in mediating SA-triggered transcriptional reprogramming.

As NPR1 itself does not have domains directly involved in DNA binding, RNA polymerase II recruitment, or chromatin modification, it might act together with other transcriptional regulators (11,12). The TGACG-motif binding proteins (TGAs) are basic leucine-zipper (bZIP) transcription factors, and several TGAs physically interact with NPR1 to induce *PR* gene expression (13–16). NPR1 forms a complex with CBP/p300-family histone acetyltransferases, HISTONE ACETYLTRANSFERASE OF THE CBP FAMILY1 (HAC1) and HAC5 (HAC1/5), and the complex is then recruited to *PR* genes via TGAs upon SA signaling to induce histone H3 acetylation (H3Ac)-mediated gene activation (3). Furthermore, 21% of INA- and *NPR1*-dependently expressed genes are regulated in an *HAC1/5*-dependent manner (3). Thus, TGAs and HACs seem to be part of components that confer DNA-binding and co-activator functions, respectively, to NPR1.

Despite the indispensable role of NPR1 in SA-triggered immunity, genome-wide direct targets of NPR1 that are specific to the SA signal have not been fully identified. Genome-wide targets of NPR1, identified after co-treating *Arabidopsis thaliana* plants constitutively overexpressing *NPR1* with SA and jasmonic acid (JA), were recently reported (17). In contrast to SA, which induces resistance against biotrophic and hemi-biotrophic pathogens, JA activates defense against necrotrophic pathogens and insects and acts antagonistically to SA in plant immunity (1). SA-specific genome-wide direct targets of NPR1 identified under a native *NPR1*-expressing condition are yet to be reported.

In this study, we identified INA (a functional SA analog)-specific genome-wide NPR1 targets using Arabidopsis expressing *NPR1* under its native promoter. Through comparative analyses with RNA-seq data showing INA- and *NPR1*-dependently expressed genes, we demonstrate that NPR1 primarily targets and activates transcription factor-encoding genes in an INA-dependent manner, triggering transcriptional cascades during INA-induced immunity. Furthermore, we report genome-wide co-targets of NPR1 and HAC1 and show that the co-targeting activity of NPR1 and HAC1 is essential for INA-dependent H3Ac and expression of a subset of NPR1 target genes. Our study reveals that the TGACG motif is abundant in NPR1-targeting regions and that TGA2 targets regions containing this motif. Finally, we report a group of genes already targeted by NPR1 in the basal state, which is enriched with genes encoding transcription factors and shows more rapid and robust induction upon SA treatment compared to genes targeted by NPR1 only after SA signaling.

## Materials and methods

### Plasmid construction

For the construction of *pHAC1::HAC1:mCherry*, a *HAC1* genomic DNA harboring ∼550 bp upstream promoter region was amplified by PCR with HAC1 promoter-F and HAC1-R (w/o stop) primer pairs (Supplementary Table S1). The PCR product was inserted into pENTR/SD/D-TOPO plasmid (Invitrogen, K242020) and then transferred into pEarleyGate 301 plasmid by recombination using Gateway LR clonase II (Invitrogen, 11791–020). For the construction of *pTGA2::TGA2:mCherry*, the *TGA2* coding region was amplified by PCR using Nde I-TGA2-ORF-F and TGA2-ORF-R (w/o stop) primers (Supplementary Table S1) and cloned into pENTR/SD/D-TOPO plasmid. For *TGA2* promoter cloning, a genomic DNA containing ∼1.5 kb promoter region of *TGA2* was amplified by PCR with Not I-TGA2 promoter-F and Nde I-TGA2 promoter-R primers (Supplementary Table S1) and then inserted into Not I and Nde I sites within the plasmid with cloned TGA2-coding region. The resulting *TGA2* construct composed of the promoter and coding region was transferred into pEarleyGate 301 plasmid by recombination. Finally, the HA tag within the *HAC1*- and *TGA2*-containing pEarleyGate 301 plasmids was replaced by mCherry tag derived from pGGC015 (18) plasmid by using Asc I and Pac I sites.

### Plant materials and growth conditions

*Arabidopsis thaliana* accession Columbia-0 (Col) was used as a genetic background for all experiments in this study. *pHAC1::HAC1:mCherry* transgenic plant was generated by the transformation of *hac1-2* (SALK_070277) with *pHAC1::HAC1:mCherry* plasmid. *pTGA2::TGA2:mCherry* transgenic plant was generated by introducing *pTGA2::TGA2:mCherry* plasmid into the *tga2 tga5 tga6* triple mutants. The *tga2 tga5 tga6* mutant was described previously (19). Floral dip method via *Agrobacterium tumefaciens* strain GV3101 was used for plant transformation. The *pNPR1::NPR1:GFP npr1-1* transgenic plant (3) and *npr1-1* mutant (5) were previously described. For chromatin immunoprecipitation (ChIP) assays, plants were grown on Murashige and Skoog (MS) basal medium under 8-hour (h) light/16-h dark (8L/16D) photoperiod for 4 weeks (w) at 22°C. For RNA extraction and RT-qPCR assays, plants were grown on soil under 8L/16D photoperiod for 6 w at 22°C. For 2,6-dichloroisonicotinic acid (INA) treatment, plants were sprayed with distilled water (−INA) or 300 μM INA (+INA; Sigma-Aldrich 456543) and then incubated under constant light for 12 h before harvesting.

### Chromatin immunoprecipitation (ChIP) assays

4-w-old seedlings were infiltrated with 1% formaldehyde solution under vacuum for cross-linking. The cross-linking was then quenched by adding glycine to final 125 mM and applying vacuum. After grinding the cross-linked seedlings to fine powder, nuclei were extracted by following the protocol of Saleh et al. (20) with minor modifications. Briefly, ground powder was suspended in 25 ml of nuclei isolation buffer (0.25 M sucrose, 15 mM PIPES pH 6.8, 15 mM NaCl, 5 mM MgCl_2_, 60 mM KCl, 1 mM CaCl_2_, 9% Triton X-100, 1 mM PMSF, 0.05 μg/ml antipain, 0.5 μg/ml bestatin, 0.5 μg/ml leupeptin, and 4 μg/ml pepstatin) and incubated on ice for 10 minutes (min). Then, the suspension was filtered twice through two-layered miracloth and centrifuged at 9050 g for 20 min at 4°C. For chromatin digestion using micrococcal nuclease (MNase), nuclei pellet was resuspended in 700 μl of MNase working buffer (50 mM HEPES pH 7.5, 3 mM CaCl_2_, 1 mM PMSF, 100 μM MG132, and protease inhibitor cocktail) with MNase (NEB, M0247S) at 10 000 gel units/ml. MNase digestion was performed at 25°C for 15 min followed by at 28°C for 5 min. 5xMNase stop buffer (50 mM HEPES pH 7.5, 50 mM EDTA, 0.5% SDS, 1 mM PMSF, and protease inhibitor cocktail) was then added, and the solution was incubated on ice for 10 min. To extract chromatin from nuclei, sonication was performed with 15 cycles of 5-second (sec) on/10-sec off pulse at 15% amplitude using Sonic Dismembrator 500 (Fisher Scientific, USA, 15–338-550). After centrifugation at 13 000 rpm for 10 min, the supernatant was diluted 5 folds with ChIP dilution buffer (20 mM HEPES pH 7.5, 187.5 mM NaCl, 7% sucrose, 0.625% Triton X-100, 1 mM PMSF, 0.05 μg/ml antipain, 0.5 μg/ml bestatin, 0.5 μg/ml leupeptin, and 4 μg/ml pepstatin). Subsequently, the lysate was precleared by adding 60 μl of protein A agarose beads (Santa Cruz, sc-2001) and incubated with rotation at 4°C for 1 h. For immunoprecipitation, GFP- or RFP-trap agarose beads (Chromotek, gta-20 or rta-20, respectively) were added to the lysate after the removal of protein A agarose beads by centrifugation. After overnight incubation at 4°C, GFP- or RFP-trap agarose beads within the lysate were then collected by centrifugation and washed as following: (i) Once with low salt wash buffer (20 mM Tris–HCl pH 8.0, 150 mM NaCl, 2 mM EDTA, 0.2% SDS, and 0.5% Triton X-100), (ii) once with high salt wash buffer (20 mM Tris–HCl pH 8.0, 500 mM NaCl, 2 mM EDTA, 0.2% SDS, and 0.5% Triton X-100), (iii) once with LiCl wash buffer (10 mM Tris–HCl pH 8.0, 0.25 M LiCl, 1 mM EDTA, 0.5% NP-40, and 0.5% sodium deoxycholate) and (iv) twice with TE buffer (10 mM Tris–HCl pH 8.0 and 1 mM EDTA). Next, immunoprecipitated DNA-protein complexes were eluted using 300 μl of elution buffer (1% SDS and 100 mM NaHCO_3_) at 65°C for 20 min with high-speed agitation. Then, the eluate was incubated at 65°C for at least 6 h in the presence of 200 mM NaCl to reverse cross-linking. Proteins separated from DNA within the eluate were cleaved by using proteinase K (Roche, 03 115 828 001). Finally, DNA was purified using QIAquick PCR Purification Kit (Qiagen, 28106).

ChIP assays involving sonication for chromatin shearing were performed essentially as described by Saleh et al., (20) with the following minor modifications. Nuclei, which were extracted as above, were resuspended in 1 ml of nuclei lysis buffer (50 mM HEPES pH 7.5, 150 mM NaCl, 1 mM EDTA, 1% SDS, 0.1% sodium deoxycholate, 1% Triton X-100, 1 mM PMSF, 0.05 μg/ml antipain, 0.5 μg/ml bestatin, 0.5 μg/ml leupeptin, and 4 μg/ml pepstatin) and incubated on ice for 10 min. Then the lysate was divided into two equal-volume aliquots, and chromatin within the aliquots was sheared by sonication with 9 cycles of 15-s on/1-min off pulse at 33% amplitude using Sonic Dismembrator 500 (Fisher Scientific, USA, 15–338-550). After centrifugation, combined supernatant from the aliquots was diluted 5-fold with the nuclei lysis buffer described above. The rest of procedures was the same with the one for MNase digestion.

### ChIP quantitative PCR (ChIP-qPCR) assays

The amount of DNA obtained from ChIP was measured by qPCR with primers listed in Supplementary Table S2. The 2^−ΔΔ^*^C^*_T_ method (21) was used to calculate the relative amount of amplified DNA in sample. The value of product amplified from each IP sample was normalized to the values generated from the respective input DNA and *Actin 2* (*ACT2*) to assess enrichment levels.

### ChIP-sequencing (ChIP-seq)

For DNA collected from ChIP involving MNase digestion, sonication was additionally performed with 14 cycles of 30-sec on/30-sec off pulse using Bioruptor Pico (Diagenode) to maximize the production of DNA in the size range proper for sequencing. ChIP DNA libraries were generated by using NEBNext® Ultra™ II DNA Library Prep with Sample Purification Beads (NEB, E7103) and NEBNext® Multiplex Oligos for Illumina® (NEB, E7335) following the supplier's instruction. ChIP-seq with 101-bp paired-end reads was performed on Illumina HiSeq 4000.

### Next-generation sequencing (NGS) data analysis

NGS data analyses were mainly performed on the public server at the Galaxy (https://usegalaxy.org/) with the following details. Reads generated from ChIP-seq were first trimmed by using Trimmomatic (22) with options of “-phred33 ILLUMINACLIP:TruSeq3-PE.fa:2:30:10 LEADING:3 TRAILING:3 SLIDINGWINDOW:4:15 MINLEN:36”. Read mapping was then performed on Bowtie (23) with options of “-S –best –strata -X 500 -m 1 –chunkmbs 500” to align the reads to the TAIR10 Arabidopsis genome. Sequence alignment/map (SAM) files generated through read mapping were converted to binary alignment/map (BAM) files by using SAMtools (24). Subsequently, peak calling was executed using Model-based Analysis of ChIP-Seq (MACS2) (25) with options of “-f BAMPE -g 1.10e8 –bw 300 –mfold 10100 -q 0.05”. Input data were used as controls for peak calling. Differential peaks between genotypes and/or treatments were identified by using MACS2 bdgdiff with options of log_10_ likelihood ratio cutoff 0.5 and minimum length 100. By intersecting differential peaks between two biological replicates using BEDTools (26), overlapping peaks were identified and finally determined as NPR1:GFP- or HAC1:mCherry-peaks. Likewise, common peaks between NPR1:GFP- and HAC1:mCherry-peaks were determined by identifying overlapping peaks in the same way. Next, binding peaks were annotated with the gene that contains the transcriptional start site (TSS) closest to each binding peak using ChIPseeker (27). Genomic regions located 3 kb upstream from the TSS to the TSS itself were defined as TSS regions. TSS regions within 1 kb upstream of the TSS were classified as promoters, whereas those located within 1–2 kb or 2–3 kb upstream of the TSS were designated as upstream regions. To visualize sequence reads using the integrative genomics viewer (IGV), bigwig files were generated as following: BAM files were deduplicated using Picard markDuplicate, and then the deduplicated BAM files were converted to bedgraph files using bedtools genomecov. Finally, bedgraph files were converted to bigwig files using bedGraphToBigWig. Enrichment scores were calculated as following: Using BamCompare, all ChIP-seq reads were normalized to reads per kilobase of bin per million mapped reads (RPKM) values with a bin size of 5 bp, and the RPKM values derived from an IP sample were then subtracted by the RPKM values derived from the corresponding input sample to obtain enrichment scores. Next, average enrichment scores between two biological repeats were calculated on BigwigCompare with bigwig files generated from BamCompare with option of bin size 5. Enrichment scores for the selected genomic regions were then calculated by using ComputeMatrix with option of bin size 5. PlotHeatmap was used to visualize enrichment scores within genomic regions enriched with NPR1:GFP or HAC1:mCherry. DNA motif sequences enriched in binding peaks were predicted by using Multiple Em for Motif Elicitation (MEME; https://meme-suite.org/meme/ (28) with options of “-mod zoops –minw 6 –maxw 10 -markov_order 1”.

Histone H3 acetylation (H3Ac) ChIP-seq and RNA-seq data used in this study were obtained from our previous study (3). Normalization of H3Ac ChIP-seq reads and calculation of H3Ac enrichment scores were performed as described above, and profile plots for H3Ac enrichment were generated by using PlotHeatmap. To visualize sequence reads of RNA-seq as IGV snapshots, bigwig files of RNA-seq were generated from BAM files on BamCoverage with options of “–bs 10 –normalizeUsing RPKM”.

To analyze the transcriptomes of 1 mM SA-treated WT Col, we downloaded the raw RNA-seq data (BioProject ID PRJNA224133; (29) from the Short Read Archive (https://www.ncbi.nlm.nih.gov/sra/). The data of two biological replicates each including four technical runs were analyzed as following: Fastq files were first trimmed by using Trimmomatic with default parameters. The trimmed reads were then aligned to the TAIR10 Arabidopsis genome by using Bowtie2 with default sets, generating four BAM files from four technical runs of each sample. After merging BAM files, read counting was performed on HTSeq-count (30) with options of “-m intersection-strict –a 10”. For differential expression analysis between SA- and mock-treatment samples, DESeq2 (31) with parameters of log_2_ fold change (FC) ≥ 1 and *P*-value < 0.05 was used with the count data of two biological replicates to obtain differentially expressed genes (DEGs) between the two samples.

Pheatmap R package was used to visualize gene expression levels. Gene ontology (GO) enrichment analysis was performed at the database for annotation, visualization, and integrated discovery (DAVID) (https://david.ncifcrf.gov/; (32). GO terms satisfied with FDR < 0.05 were visualized using ggplot2 R package. To construct and visualize a network of gene-sets within GO terms, Enrichment Map (33) was used with options of *P*-value <0.005, *Q*-value <0.05, and Jaccard Overlap combined coefficient >0.25 with combined constant = 0.15 or 0.25. The network was then clustered by using AutoAnnotate (34), and the label of each cluster was manually edited.

### RNA extraction and RT-qPCR assays

Total RNA was extracted using TRI reagent (Molecular Research Center TR118) from leaves of 6-w-old plants. Reverse transcription was carried out using 3 μg of total RNA with RevertAid reverse transcriptase (Thermo Scientific EP0442), followed by real-time quantitative PCR (qPCR) analyses. The RT-qPCR was performed in the Rotor-Gene Q 2plex real-time PCR system (QIAGEN) using TOPreal qPCR 2X PreMIX (SYBR Green with low ROX) (Enzynomics RT500). The values obtained from cDNA amplification were normalized to the expression of the housekeeping gene *ubiquitin10* (*UBQ10*) and presented as transcript levels relative to the transcript level of the corresponding gene in wild-type plants that were not treated with INA. To calculate relative transcript levels, the transcript level of the wild-type plant not treated with INA was set to 1. The sequences of primers used for RT-qPCR are listed in Supplementary Table S3.

### SA tolerance assays

Plants were grown on MS basal medium containing 0.05% dimethyl sulfoxide (DMSO; Kanto Chemical 10378-00) as a control or supplemented with 0.3 mM SA (Sigma-Aldrich 257588). SA was first dissolved in DMSO and then added to MS basal medium. After incubation under a 16L/8D photoperiod at 22°C for 10 days, the plants were observed with the naked eye to compare their tolerance to SA between genotypes.

### Microscopy

Confocal laser scanning microscope (Carl Zeiss LSM 700) was used to acquire images of root tips from 1-w-old Arabidopsis seedlings grown on MS basal medium under a 16L/8D photoperiod at 22°C. A 555 nm solid-state laser was used to observe mCherry-fused proteins expressed in transgenic Arabidopsis plants.

### Flowering time analyses

Flowering times were determined by counting the number of rosette leaves at the time of bolting. Plants were grown on soil under a 12L/12D photoperiod at 22°C.

## Results

### NPR1 is usually targeted to promoters or promoter-vicinity regions in an INA-dependent manner

To gain an unbiased, holistic view of the role of NPR1 in SA-induced transcriptional reprogramming required for plant immunity, we investigated genome-wide direct targets of NPR1 in Arabidopsis. For this, we performed chromatin immunoprecipitation followed by sequencing (ChIP-seq) using seedlings expressing green fluorescent protein (GFP)-fused NPR1 under a native *NPR1* promoter (*pNPR1::NPR1:GFP npr1-1*, hereafter *NPR1:GFP*) and treated or not with a functional SA analog, INA. The introduction of *pNPR1::NPR1:GFP* into *npr1-1* restored the INA-induced expression of *PR1*, an SA-responsive marker gene (Supplementary Figure S1A). In addition, the intolerance of *npr1-1* to a high level of SA was also restored by *pNPR1::NPR1:GFP* (Supplementary Figure S1B). These results indicate that NPR1:GFP protein is functionally equivalent to NPR1 protein and the ChIP-seq data obtained for NPR1:GFP are likely to represent genuine NPR1 targets. For the first two ChIP-seq biological replicates (hereafter rep1&2), we performed immunoprecipitation (IP) with 1% sodium dodecyl sulfate (SDS), which might improve the signal-to-noise ratio in peak calling and thus only detect robust binding. For IP of the other two biological replicates (hereafter rep3&4), we used 0.02% SDS to also detect weaker binding. In addition, as sonication might dissociate protein complexes, we used two different chromatin fragmentation methods: sonication for rep1&2 and chromatin digestion by micrococcal nuclease (MNase) treatment for rep3&4. Consequently, we generated two different ChIP-seq datasets, each including two biological repeats, to identify genome-wide NPR1 targets with or without INA treatment. Then, for each dataset, we called NPR1 peaks by identifying common peaks among the two biological replicates.

**Figure 1.**
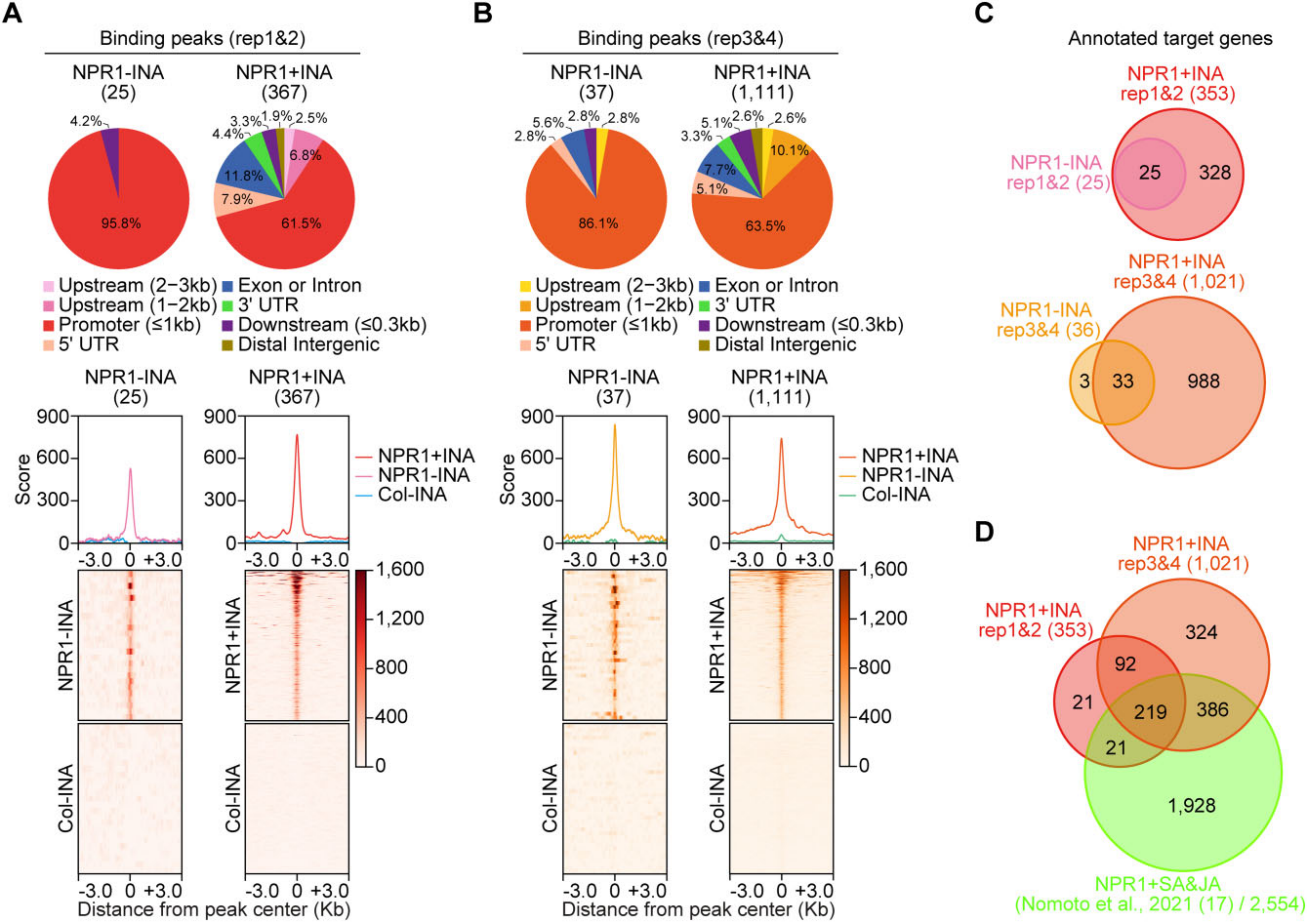
NPR1 is usually targeted to promoters or promoter-vicinity regions in an INA-dependent manner. (A, B) Genome-wide distribution and enrichment of NPR1:GFP binding peaks. Pie-charts illustrate the distribution of genomic regions enriched with NPR1:GFP. Profile plots show the average scores of NPR1:GFP enrichment in regions from the 3 kb upstream to the 3 kb downstream of NPR1:GFP-peak centers. Heatmaps visualize enrichment scores corresponding to individual peaks. NPR1:GFP peaks were identified through two biological repeats of chromatin immunoprecipitation followed by sequencings (ChIP-seqs) using *pNPR1::NPR1:GFP* transgenic (NPR1) or WT Col plants and anti-GFP antibody in the absence (NPR1 − INA or Col − INA) or presence (NPR1 + INA) of 2,6-dichloroisonicotinic acid (INA; a functional SA analog) treatment. Peak numbers are indicated in parentheses below the names of binding peaks. All enrichment scores presented as profile plots or heatmaps are the means of enrichment levels derived from two biological repeats and were compared to the enrichment scores of Col − INA, a negative control. To calculate enrichment scores, ChIP-seq reads were normalized using reads per kilobase of bin per million mapped reads (RPKM) method with a bin size of 5 bp. The RPKM values derived from each IP sample were then subtracted by the RPKM values derived from the input sample to calculate enrichment levels. The extended regions from the peak centers were equally divided into 5 bp bins. **(A)** or **(B)** presents data analyzed from the replicates 1 and 2 (rep1&2) or replicate 3 and 4 (rep3&4), respectively. The two datasets each consisting of two biological repeats were derived from ChIP-seqs performed at different experimental conditions (see Materials and Methods section). **(C)** Venn diagrams showing the numbers and overlaps between NPR1-target genes identified under − INA or + INA conditions. Total numbers of annotated targets are indicated in parentheses. **(D)** Venn diagram illustrating overlaps and differences in NPR1-target genes identified by different ChIP-seqs. The NPR1-target genes identified from our two datasets (rep1&2 and rep3&4) were also compared to the NPR1-target genes reported by Nomoto *et al.* (17), which was obtained by ChIP-seq after the co-treatment (SA&JA) of SA and jasmonic acid (JA).

To study the genome-wide distribution of NPR1, we classified genomic regions containing NPR1 peaks. In the case of the rep1&2 dataset, nearly 96% or 76% of NPR1 peaks identified without or with INA treatment, respectively, were located within the promoters or promoter-vicinity regions (Figure 1A). Likewise, nearly 89% or 79% of NPR1 peaks identified without or with INA treatment, respectively, from the rep3&4 dataset were located within the promoters or promoter-vicinity regions (Figure 1B). As expected from our experimental design, we identified more NPR1 peaks from the rep3&4 dataset (Figure 1B) than from the rep1&2 dataset (Figure 1A). Comparative analyses using enrichment scores obtained from *NPR1:GFP* and wild-type (WT) Columbia-0 (Col-0) plants showed that the identified peaks are specific to the *NPR1:GFP* plants (Figure 1A and B), confirming the reliability of our peak calling. Thus, NPR1 peaks reside usually within promoters or promoter-vicinity regions of the Arabidopsis genome.

To identify genome-wide NPR1-target genes, we assigned NPR1 peaks obtained from INA treated (+INA) or untreated (−INA) plants to the nearest genes, which we named NPR1 + INA or NPR1 − INA targets, respectively (Supplementary Dataset S1). Comparisons between NPR1 + INA and NPR1 − INA targets revealed that 93% (328/353 of the rep1&2 dataset) or 97% (988/1021 of the rep3&4 dataset) of the NPR1 + INA targets show INA-dependent NPR1-targeting activity, whereas the remaining 7% (25/353 of the rep1&2 dataset) or 3% (33/1021 of the rep3&4 dataset) show NPR1-targeting activity already in the absence of INA (Figure 1C). In addition, 88% (311/353) of the NPR1 + INA targets identified from the rep1&2 dataset were also identified as NPR1 + INA targets from the rep3&4 dataset (Figure 1D), indicating that most robust NPR1 + INA targets were reproducibly identified in the two experimental conditions. Together, these results indicate that NPR1 targeting is largely INA (SA analog) dependent.

As Nomoto *et al.* (17) recently reported the genome-wide NPR1 targets in Arabidopsis plants overexpressing *NPR1* and co-treated with SA and JA (17), we compared their data with the NPR1 targets we identified. Only 25% (626/2554) of the NPR1 targets reported by Nomoto et al. (17) were among our NPR1 + INA targets (Figure 1D). This large discrepancy is probably due to differences in the experimental conditions: Nomoto *et al.* (17) treated Arabidopsis plants overexpressing NPR1 with SA and JA for 6 h under long day (16L/8D) photoperiod, whereas in our study, we treated Arabidopsis plants expressing NPR1 under its native promoter with INA for 12 h under short day (8L/16D) photoperiod. Therefore, we identified INA-specific genome-wide NPR1 targets in cells natively expressing *NPR1*.

### INA-dependent NPR1 targeting primarily induces the transcriptional activation of genes encoding transcription factors

**Figure 2.**
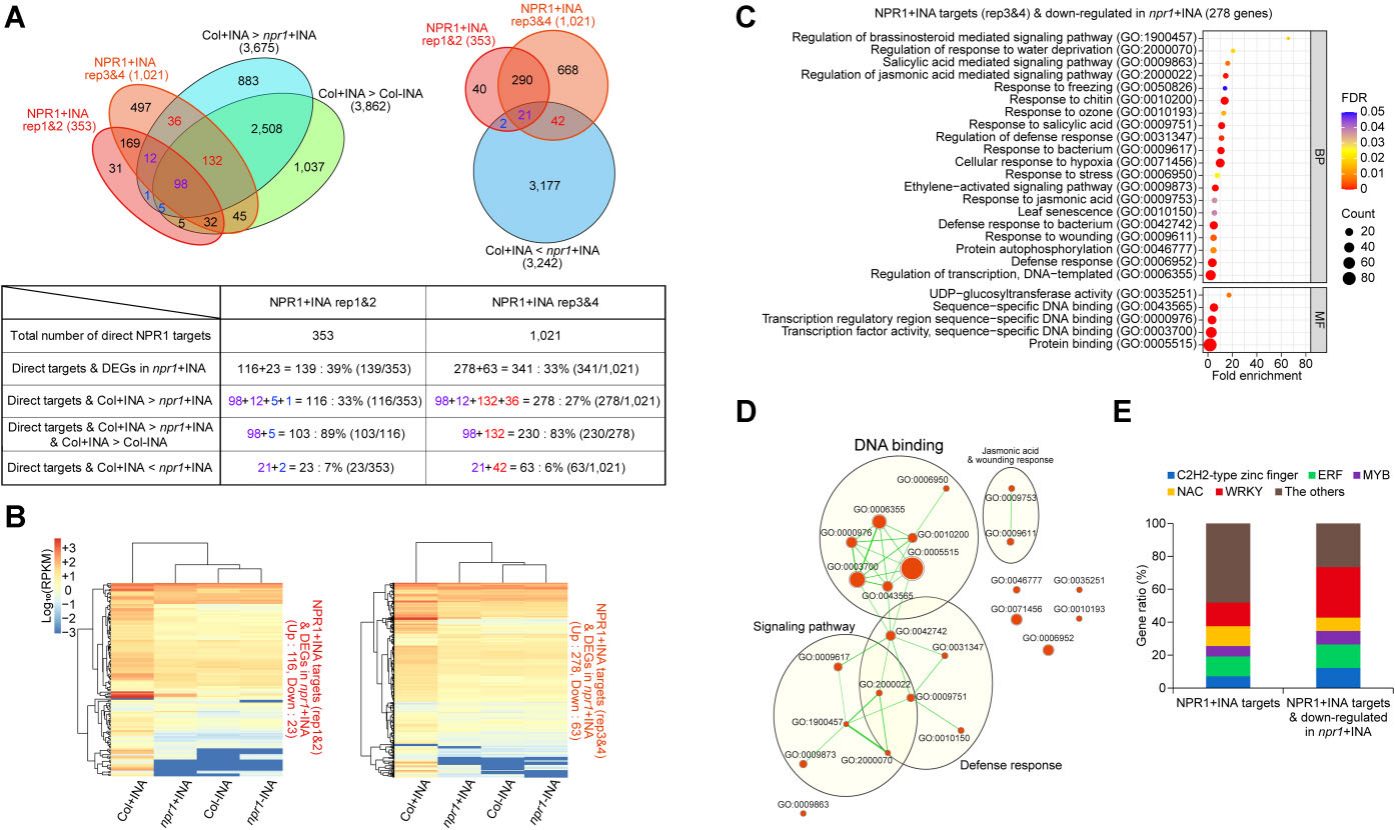
Direct NPR1 targets include hundreds of genes NPR1-dependently activated during SA-triggered immunity and are enriched mostly with DNA-binding factor encoding genes. **(A)** Venn diagrams illustrating the numbers of genes that are targeted and regulated by NPR1 after INA treatment (+INA). Genes showing *NPR1*-dependent or INA-induced expression (absolute log_2_ fold change value ≥ 1, FDR ≤ 0.2) were identified from the reported RNA-seq data (3). Total numbers of NPR1-target genes or differentially expressed genes (DEGs) are indicated in parentheses. Blue or red numbers mean the numbers of genes showing *NPR1*-dependent expression among the NPR1 targets identified from the rep1&2 or rep3&4 dataset, respectively, whereas the numbers of genes co-identified from both datasets are marked in purple. The table below the Venn diagrams summarizes the numbers of NPR1-target genes showing *NPR1*-dependent up- and/or down-regulation along with the percentages of each subsets. **(B)** Heatmaps showing the expression levels of genes that are directly regulated by NPR1 upon INA treatment. Expression levels are presented as log_10_ values of reads per kilobase of transcript per million mapped reads (RPKMs). Hierarchical cluster analysis between genotype and/or treatment was performed based on similarity of gene expression. **(C)** Gene ontology (GO) terms enriched among the genes that are targeted and directly activated by NPR1 upon INA treatment. Two GO categories are indicated in the grey boxes (BP; biological process, MF; molecular function). The enriched GO terms were selected with cutoff of FDR < 0.05. NPR1 targets within the rep3&4 dataset of ChIP-seq were analyzed. **(D)** Enrichment map visualizing the networks and clusters of gene sets obtained from the GO analysis in **(C)**. Each node represents an enriched GO term. Node size or edge width is proportional to the number of genes within the node or shared between two connected nodes, respectively. The gene sets were selected with Q-value < 0.05, and the edge cutoff meaning a similarity between a pair of gene sets was 0.25 with 0.25 of Jaccard and overlap combined constant. Representative biological or molecular functions of clustered gene sets are highlighted. The font size of cluster label is proportional to the cluster size. **(E)** Stacked bar chart indicating the proportion of each transcription-factor family among the total transcription factors of which genes are directly targeted by NPR1 upon INA treatment. Right-side bar indicates NPR1-target transcription factors that are also activated in an *NPR1*-dependent manner. Analyzed transcription factors were selected from the GO analysis in (C).

To determine the influence of NPR1 targeting on transcription at the genome level, we combined the ChIP-seq data from this study with the RNA-seq data from our previous study (3). Hundreds of NPR1 + INA target genes were *NPR1*-dependently upregulated upon INA treatment (Figure 2A and Supplementary Dataset S2). In the case of the rep1&2 dataset, 33% (116/353) of the NPR1 + INA targets were downregulated and 7% (23/353) were upregulated by the *npr1-1* mutation in the presence of INA. In addition, 89% (103/116) of the NPR1 + INA targets downregulated by *npr1-1* were induced by INA treatment in the WT. We obtained similar results from the rep3&4 dataset: 27% (278/1021) and 6% (63/1021) of the NPR1 + INA targets were downregulated and upregulated, respectively, by *npr1-1* in the presence of INA, and 83% (230/278) of the downregulated genes were induced by INA in the WT. We then analyzed the expression patterns of *NPR1*-dependently expressed NPR1 + INA targets. Most of these genes were upregulated in an *NPR1*-dependent manner under the + INA condition (Figure 2B): 83% (116/139 of the rep1&2 dataset) or 82% (278/341 of the rep3&4 dataset) of the *NPR1*-dependently expressed NPR1 + INA targets were transcriptionally activated in the presence of INA. Thus, INA-induced NPR1 targeting generally results in the transcriptional activation rather than repression of the direct targets.

Next, we functionally classified the genes that are targeted and directly regulated by NPR1 to establish the primary role of NPR1 in SA-triggered immunity at the genome level. As NPR1 targets are generally upregulated after INA treatment (Figure 2B) and NPR1 positively regulates SA-triggered immunity, we focused on the NPR1 + INA target genes showing *NPR1*-dependent upregulation by INA treatment. Gene Ontology (GO) enrichment analysis revealed that NPR1 directly activates genes involved in various phytohormone-mediated signaling pathways, responses to biotic or abiotic stresses, and transcription (Figure 2C, Supplementary Figure S2A and Supplementary Dataset S3). We then clustered the GO terms based on overlapping gene sets and found that genes encoding DNA-binding factors formed the largest group (Figure 2D, Supplementary Figure S2B, and Supplementary Dataset S3). These results indicate that NPR1 primarily activates transcription factor-encoding genes upon SA signaling, and these factors might in turn activate diverse downstream defense genes. Consistent with this idea, when we clustered GO terms for genes that are *NPR1*-dependently upregulated but not identified as direct NPR1 targets, genes related to a variety of defense-related functions except for DNA binding were classified (Supplementary Figure S2C and D; Supplementary Dataset S4). Genes involved in chloroplast activity, tissue development, and cell division were abundant among the genes indirectly downregulated by NPR1 (Supplementary Figure S2E and F; Supplementary Dataset S4). The transcription factor genes directly targeted and upregulated by NPR1 were from diverse families (Figure 2E). WRKY family members accounted for about one-third of these transcription factors. Although transcription factor-encoding genes were also abundant among NPR1 + INA targets that are not NPR1-dependently upregulated, these genes do not seem to play a role in SA-triggered immunity as evidenced by their GO terms (Supplementary Figure S3 and Supplementary Dataset S5). In summary, these results indicate that the primary role of NPR1 in INA (also probably SA)-induced transcriptional reprogramming is to directly activate the expression of transcription factor-encoding genes and thus trigger transcriptional cascades required for plant immunity.

### NPR1 directly activates genes in diverse SA-dependent immunity pathways

Although not functionally classified by our GO analyses, we also found genes involved in the SA-dependent immunity pathway among the genes directly upregulated by NPR1 (Supplementary Figure S4). Among them, *RESPIRATORY BURST OXIDASE HOMOLOG D* (*RBOHD*) encodes an NADPH oxidase that generates reactive oxygen species (ROS) upon pathogen attack (35,36), and *ATP-BINDING CASSETTE G40* (*ABCG40*) encodes a transporter responsible for abscisic acid (ABA) import-mediated stomatal closure to restrict pathogen entry (37). We also found genes encoding calmodulin domain-containing protein kinases, and one of these kinases, CALMODULIN-DOMAIN PROTEIN KINASE5 (CPK5), interacts with and phosphorylates LYSM-CONTAINING RECEPTOR-LIKE KINASE5 (LYK5), a major chitin receptor, to activate downstream immunity signaling pathways (38). CPK5 also directly phosphorylates WRKY33 and increases its DNA-binding ability, contributing to camalexin (an antimicrobial substance) biosynthesis (39).

We also identified genes that encode regulators of effector-triggered immunity (ETI) among the direct NPR1 activation targets. Among them, we found *RPM1-INDUCED PROTEIN KINASE* (*RIPK*), a receptor-like cytoplasmic kinase gene, which encodes a protein kinase that enables RESISTANCE TO P. SYRINGAE PV MACULICOLA1 (RPM1) to recognize the bacterial effectors AvrB and AvrRpm1 and triggers RPM1-dependent immunity (40). In addition, *UDP-GLUCOSYL TRANSFERASE 73B3* (*UGT73B3*) and *UGT73B5* are directly activated by NPR1, and their protein products detoxify secondary metabolites accumulated after infection by bacteria harboring AvrRpm1 and consequently modulate redox-sensitive signaling pathways (41). We also found *SA-INDUCED LEGUME LECTIN-LIKE PROTEIN1* (*SAI-LLP1*), which encodes a protein that positively regulates ETI induced by AvrRpm1 and systemic acquired resistance (42,43). These examples indicate that NPR1 not only triggers transcriptional cascades but also directly regulates diverse genes involved in SA-induced immunity.

### INA-dependent NPR1 targeting is principally mediated by TGACG (TGA) motif-binding transcription factors

**Figure 3.**
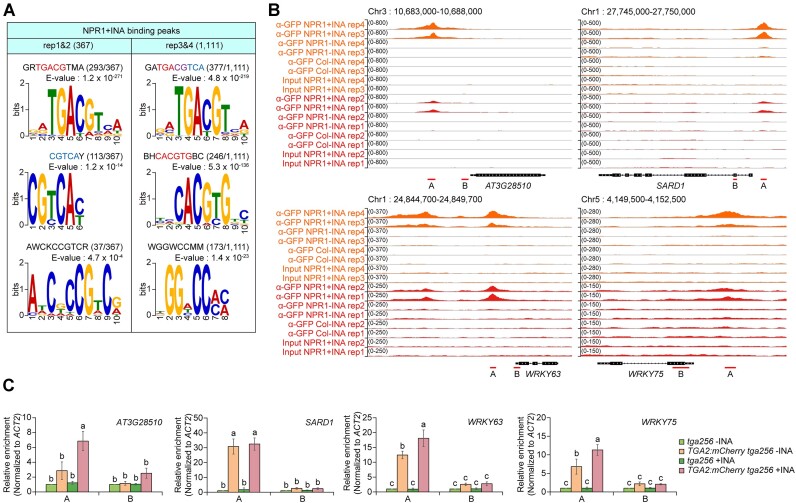
DNA motif with TGACG sequence is enriched in NPR1-binding regions, and TGA2 is targeted to the motif-containing regions. **(A)** DNA sequences enriched in INA-dependent NPR1-targeting regions. The top 3 results are displayed in descending order of E-value. The defined motif sequences are shown above E-values. Well-known motif sequences are in red for forward orientation or in blue for reverse orientation. Purple indicates overlaps between sequences colored in red and blue. Numbers of each motif occurrence are indicated in parentheses in comparison to the total numbers of input sequences. Motif analysis was performed using the MEME with option of zero or one occurrence per sequence and 1st order Markov background model. DNA sequences within regions from the 250 bp upstream to the 250 bp downstream of NPR1-binding peak centers were used for analysis. **(B)** Integrative Genomics Viewer (IGV) snapshots of NPR1 ChIP-seq data for representative NPR1-target loci containing the TGACG motif. Red lines below gene models marked with A or B indicate NPR1-binding peaks with the TGACG motif or regions distant from the peaks, respectively. Data scales are indicated in parentheses on the right side of y-axes. Chromosome (Chr) numbers and genomic regions are shown at the top of images. **(C)** TGA2-targeting activity to the NPR1-targeting regions containing the TGACG motif (regions A) or distant regions (regions B) with (+INA) or without (−INA) INA treatment. A and B regions indicated in **(B)** were amplified in ChIP-quantitative PCR (ChIP-qPCR) assays. To calculate relative enrichments, the values of *tga256*− INA were set to 1 after normalization by input and *actin2* (*ACT2*). Means ± SE of three biological replicates are shown. Two-way ANOVA analysis with Tukey's Honest Significant Differences (HSD) test was performed. Different letters above each bar mean statistically significant differences (*P*-value < 0.05).

As NPR1 does not have a DNA-binding domain, NPR1 targeting must be mediated by transcription factors. To search for candidate transcription factors capable of recruiting NPR1 onto chromatin, we performed a motif analysis on DNA sequences within regions 250 bp upstream to 250 bp downstream of the NPR1 + INA peak centers by using MEME (28) (Figure 3A). Motif analysis with the rep1&2 dataset identified only the TGACG motif with a greater frequency than the number of NPR1-binding peaks (406 versus 367), highlighting the dominance of the TGACG motif within the NPR1 peaks. Motif analysis using the rep3&4 dataset revealed the TGACG motif as the most abundant DNA sequence and the CACGTG (G-box) and WGGWCCMM sequences (putative TCP-binding motif; (44) were also identified within the NPR1 peaks. As the IP condition used for the rep1&2 dataset (with 1% SDS) was harsher than that used for the rep3&4 dataset (with 0.02% SDS), these results indicate that NPR1-targeting factors have higher affinity for chromatin harboring the TGACG motif than chromatin harboring the other transcription factor-binding motifs.

Because the TGACG motif is targeted by bZIP family TGA transcription factors and several TGA transcription factors interact with NPR1 (3,13–16), we examined if the well-known TGA transcription factor TGA2 targets to several NPR1 peaks containing the TGACG motif by ChIP-quantitative PCR (ChIP-qPCR) assays using mCherry-fused TGA2 (TGA2:mCherry) (Figure 3B and C). *pTGA2::TGA2:mCherry* in *tga2 tga5 tga6* (*tga256*) triple mutant background (*TGA2:mCherry*) partially rescued the intolerant phenotype of *tga256* to continuous SA treatment (Supplementary Figure S1B and C), indicating that TGA2:mCherry protein used for ChIP has a comparable function to native TGA2 protein. TGA2:mCherry targeted to the TGACG motif-containing NPR1 peaks in an INA-independent manner but not to regions distant from the peaks. We also verified the INA-dependent targeting activity of NPR1 at the regions targeted by TGA2:mCherry by ChIP-qPCR assays (Supplementary Figure S5). In addition, TGA2:mCherry also targeted to NPR1 peaks containing the G-box but not the TGACG motif (Supplementary Figure S6). Although more direct demonstrations might be needed, these results altogether suggest that TGA transcription factors are likely the most important mediator of NPR1 targeting at the genome level.

### INA-dependent co-targeting of HAC1 to several hundred NPR1 targets induces transcriptional activation of a subset of NPR1 target genes

We previously demonstrated that NPR1 recruits HAC1 to the *PR1* promoter by forming a transcriptional co-activator complex upon INA treatment (3). As a CBP/p300-family histone acetyltransferase, HAC1 may facilitate gene activation by loosening chromatin through H3Ac. HAC1 and its homolog HAC5 affect the expression of 21% of genes that show INA- and *NPR1*-dependent expression (3). These results suggest that NPR1 and HAC1 might co-target other genomic loci besides *PR1* during INA-induced transcriptional reprogramming.

**Figure 4.**
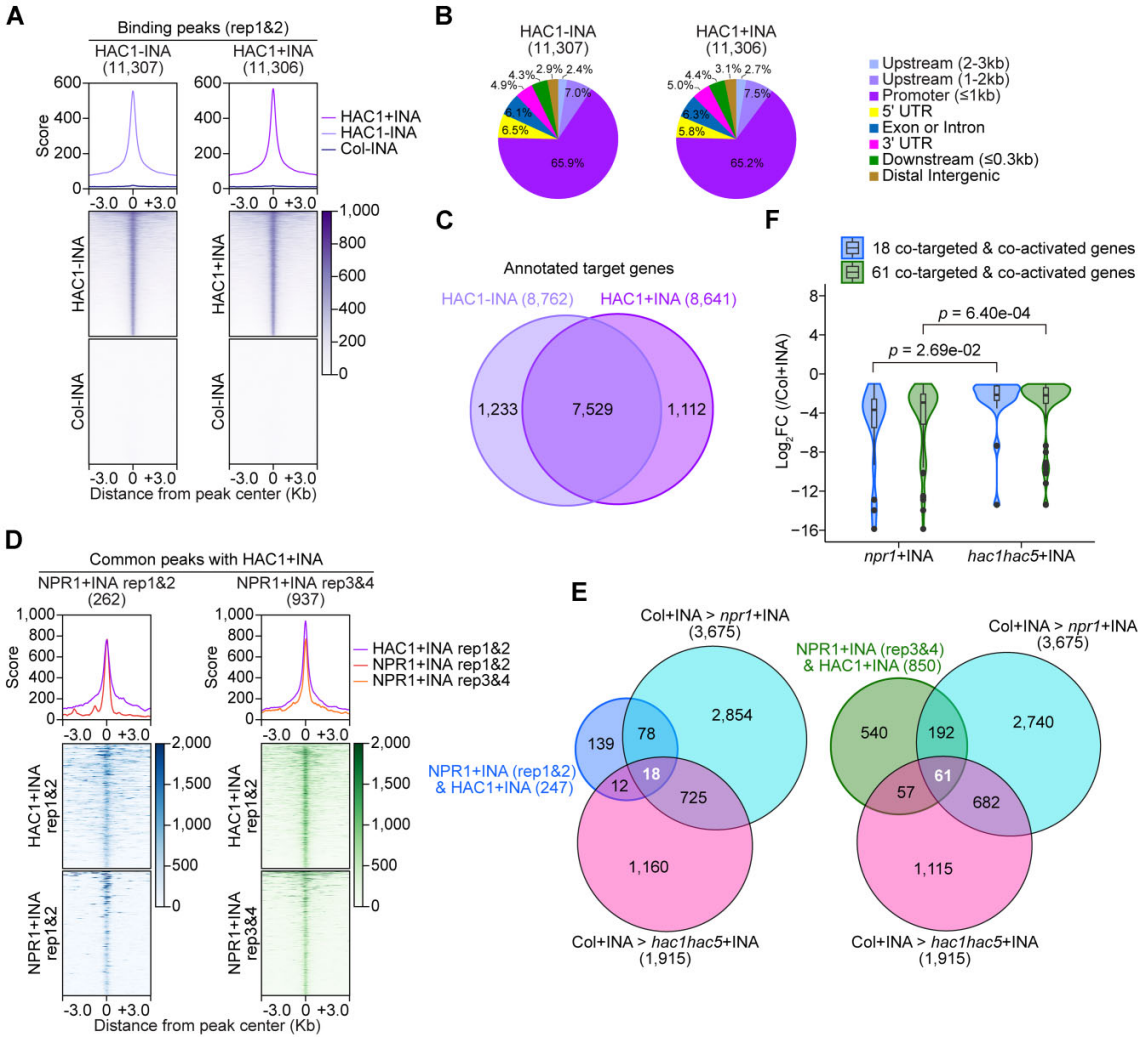
Hundreds of genes are co-targeted by NPR1 and HAC1, and this co-targeting activity is required for the transcriptional activation of a subset of the genes upon INA treatment. **(A)** Enrichment scores of HAC1:mCherry in the absence (−INA) or presence (+INA) of INA. Profile plots show average scores of HAC1:mCherry enrichment in regions from the 3 kb upstream to the 3 kb downstream of HAC1:mCherry-peak centers. Heatmaps visualize enrichment scores corresponding to individual peaks. HAC1:mCherry peaks were identified through two biological repeats of ChIP-seqs using the same conditions as the rep3&4 of NPR1:GFP ChIP-seqs. See Figure 1A, B legend for more details. **(B)** Pie-charts illustrating the distribution of genomic regions enriched with HAC1:mCherry in the absence or presence of INA. **(C)** Venn diagram showing the numbers and overlaps between HAC1-target genes identified under −INA or +INA conditions. Total numbers of annotated targets are indicated in parentheses. **(D)** Enrichment scores of the common peaks of NPR1 and HAC1 in the presence of INA. Profile plots show the average scores of enrichments in the surrounding regions harboring the common peaks of NPR1 and HAC1. Heatmaps visualize enrichment scores of NPR1 and HAC1 in individual genomic regions. The rep1&2 or rep3&4 dataset of NPR1:GFP ChIP-seq was used to obtain common peaks with the rep1&2 dataset of HAC1:mCherry ChIP-seq, respectively. Numbers of common peaks are indicated in parentheses above the profile plots. All enrichment scores are presented as the means of enrichment levels derived from two biological repeats. To calculate enrichment scores, ChIP-seq reads were normalized using RPKM method with a bin size of 5 bp. The RPKM values derived from each IP sample were then subtracted by the RPKM values derived from the corresponding input sample to calculate enrichment levels. Regions from the 3 kb upstream to the 3 kb downstream of the common-peak centers were analyzed. The extended regions from the peak centers were equally divided into 5 bp bins. **(E)** Venn diagrams illustrating the numbers of NPR1 and HAC1 co-targets showing *NPR1*- and *HAC1 HAC5*-dependent expression in the presence of INA. The co-targets were identified by annotation of the common peaks presented in **(D)**. Genes downregulated in *npr1-1* or *hac1-2 hac-5–2* mutants compared to WT Col in the presence of INA were identified from the reported RNA-seq data (3) (absolute log_2_ fold change value ≥ 1, FDR ≤ 0.2). The numbers colored white indicate the numbers of genes that are directly co-targeted and co-activated by NPR1 and HAC1 in the presence of INA. **(F)** Violin plot with included box plot showing the effects of *npr1* or *hac1 hac5* mutations on the expression of co-targets. The genes that are directly co-targeted and co-activated by NPR1 and HAC1 as identified in (E) were used for analysis. From RNA-seq data, RPKMs in *npr1-1* or *hac1-2 hac5-2* mutants were divided by RPKMs in WT (Col). Log_2_ values of the calculated fold change (FC) are presented. *P*-values shown were calculated using Wilcoxon signed rank test.

To investigate the genomic distribution and targeting activity of HAC1, we performed HAC1 ChIP-seq in the absence (−INA) or presence (+INA) of INA using seedlings expressing *mCherry*-fused *HAC1* under a native *HAC1* promoter (*HAC1:mCherry*). *HAC1:mCherry* completely rescued the phenotypes of *hac1-2* mutants such as smaller and wrinkled leaves and late flowering (Supplementary Figure S1D–F), indicating that HAC1:mCherry protein is functionally equivalent to native HAC1 protein. Therefore, our ChIP-seq data obtained by using *HAC1:mCherry* plants can be considered to represent genome-wide HAC1 targets. We performed two biological replicates of HAC1:mCherry ChIP-seq for −INA and +INA samples using the same method as used for the rep3&4 of NPR1:GFP ChIP-seq. After peak calling, we designated common peaks between the two biological replicates as HAC1-binding peaks. Comparative analyses using enrichment scores obtained from *HAC1:mCherry* and WT (Col-0) plants showed that the HAC1 peaks identified were specific to *HAC1:mCherry* (Figure 4A), demonstrating that our peak calling was accurate. We found that 79% of the HAC1 peaks located at promoters or promoter-vicinity regions (Figure 4B), consistent with the idea that HAC1 mainly affects transcription. We then annotated the HAC1 peaks obtained from −INA or +INA *HAC1:mCherry* samples to the nearest genes to identify HAC1 − INA or HAC1 + INA target genes, respectively (Figure 4C and Supplementary Dataset S6). When we compared the two target groups, 87% (7529/8641) of the HAC1 + INA targets overlapped with the HAC1 − INA targets, while only 13% (1112/8641) of the HAC1 + INA targets and 14% (1233/8762) of the HAC1 − INA targets were exclusively present in either the +INA or −INA condition, respectively (Figure 4C). Thus, these results indicate that the HAC1 peaks usually reside within promoter or promoter-vicinity regions, and, unlike NPR1 with largely INA-dependent targeting (Figure 1C), HAC1 targeting at the whole-genome level is mainly INA independent.

Next, to understand the roles of NPR1 and HAC1 during SA-induced transcriptional reprogramming, we identified genome-wide common peaks of NPR1 and HAC1 under the +INA condition (Figure 4D and Supplementary Dataset S7). Enrichment scores of both NPR1:GFP and HAC1:mCherry were the highest at the center of the common peaks (Figure 4D), indicating that we appropriately determined the common peaks. The common peaks accounted for 71% (262/367 of the rep1&2 dataset) or 84% (937/1111 of the rep3&4 dataset) of the NPR1 peaks identified under the + INA condition (Figures 1A, B, and 4D). Consistent with the HAC1-targeting activity which is mostly independent of INA, several hundred HAC1 − INA peaks were found among the NPR1 + INA and HAC1 + INA common peaks (Supplementary Figure S7A and B). 19 of the HAC1 − INA peaks were also found among the 37 NPR1 − INA peaks of the rep3&4 dataset (Supplementary Figure S7C). In addition, most of the genomic regions pre-targeted by NPR1 before INA treatment (NPR1 − INA peaks) were included within the NPR1 + INA and HAC1 + INA common peaks (Supplementary Figure S7D). Therefore, NPR1 generally targets together with HAC1 to several hundred promoter regions during INA (also probably SA)-induced transcriptional reprogramming. These results are consistent with our previous study (3), which revealed the genome-wide roles of HAC1/5 and NPR1 and the formation of the HAC-NPR1-TGA complex during SA-triggered immunity.

We then examined if NPR1 and HAC1 co-targeting activates the transcription of the common target genes. To analyze the transcriptomic changes caused by *npr1* or *hac1 hac5* mutations, we used RNA-seq data generated in our previous study (3), which also showed a functional redundancy between *HAC1* and *HAC5* with *HAC1* dominance. Integrating our ChIP-seq and RNA-seq data revealed that 18 or 61 of the common target genes are co-dependent on *NPR1* and *HAC1/5* for their INA-induced transcriptional activation depending on the rep1&2 or rep3&4 dataset of NPR1 + INA ChIP-seq used, respectively (Figure 4E and Supplementary Dataset S8). INA-induced upregulation of these genes was more severely disturbed by the *npr1* mutation than by the *hac1/5* mutations (Figure 4F). A GO enrichment analysis indicated that genes involved in defense responses are enriched among the common target genes requiring both NPR1 and HAC1/5 for transcriptional activation upon INA treatment (Supplementary Figure S8A and Supplementary Dataset S9). As these genes were not classified as transcriptional regulators by the GO analysis, the common target genes may regulate defense responses through mechanisms other than transcription in SA-triggered immunity. In addition, we observed increased INA-induced H3Ac around the NPR1 and HAC1 co-targeting sites in the WT but not in the *npr1* and *hac1/5* mutants (Supplementary Figure S8B and C). In summary, NPR1 and HAC1 co-target hundreds of genomic loci during INA (also probably SA)-induced transcriptional reprogramming and cooperatively increase the expression and H3Ac level of a subset of the common target genes. On the other hand, there were 177 genes that are NPR1 + INA but not HAC1 targets. 27 of the 177 genes were NPR1-dependently expressed but did not contain genes related to the SA-triggered immunity pathway (Supplementary Dataset S10).

### Colocalization of NPR1 and HAC1 onto chromatin is mainly mediated by TGA transcription factors

**Figure 5.**
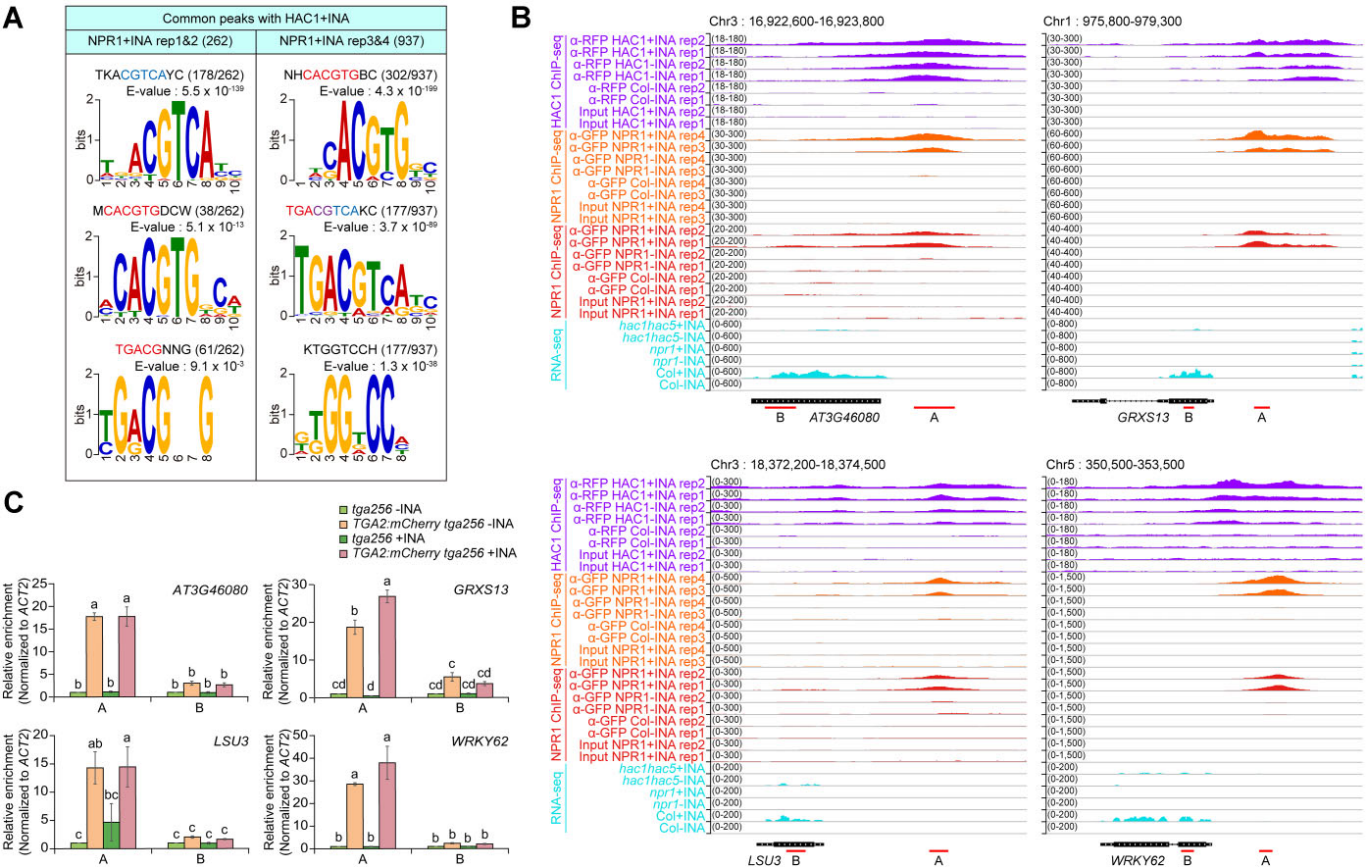
DNA motif with TGACG sequence is enriched in regions co-targeted by NPR1 and HAC1, and TGA2 is targeted to the motif-containing regions. **(A)** DNA sequences enriched in the common target regions of NPR1 and HAC1 identified in the presence of INA. The top 3 results are displayed in descending order of E-value. See Figure 3A legend for more details. **(B)** IGV snapshots of HAC1 ChIP-seq, NPR1 ChIP-seq, and RNA-seq data for the representative co-targets of NPR1 and HAC1. The representative co-targets are the genes that are activated by both *NPR1*- and *HAC1 HAC5*-dependent manners and contain TGACG motifs within the common peaks of NPR1 and HAC1. Red lines below gene models marked with A or B indicate the common peaks of NPR1 and HAC1 or regions distant from the common peaks, respectively. See Figure 3B legend for more details. **(C)** TGA2-targeting activity to the common peaks containing the TGACG motif (regions A) or distant regions (regions B) presented in (B) in the presence (+INA) or absence (−INA) of INA. A and B regions indicated in (B) were amplified in ChIP-qPCR assays. See Figure 3C legend for more experimental details.

The TGACG motif and the G-box were enriched in the NPR1 peaks, and TGA2 indeed bound to several of the TGACG motif- or G-box-containing regions (Figure 3 and Supplementary Figure S6). We then performed DNA-sequence analysis of the co-targeting sites of NPR1 and HAC1. Again, the TGACG motif and the G-box were most abundant at the co-targeting sites (Figure 5A). We tested if TGA2 is also enriched at these co-targeting sites (Figure 5B and Supplementary Figure S9A) by ChIP-qPCR using *TGA2:mCherry* transgenic plants, and TGA2:mCherry was enriched at the co-targeting sites independently of INA treatment, but not in regions distant from the co-targeting sites (Figure 5C and Supplementary Figure S9B). At these regions targeted by TGA2:mCherry, we could verify the co-targeting activities of NPR1:GFP and HAC1:mCherry in the presence of INA by ChIP-qPCR assays (Supplementary Figure S9C and D). These results are consistent with our previous study that showed INA-independent targeting of TGA2 and INA-dependent formation of the HAC–NPR1–TGA complex at the *PR1* promoter (3) and suggest that the HAC–NPR1–TGA complex might be targeted to hundreds of TGACG motif- or G-box-containing loci, mostly in an INA-dependent manner.

### Pre-targeting of NPR1 results in more rapid and robust induction by SA

**Figure 6.**
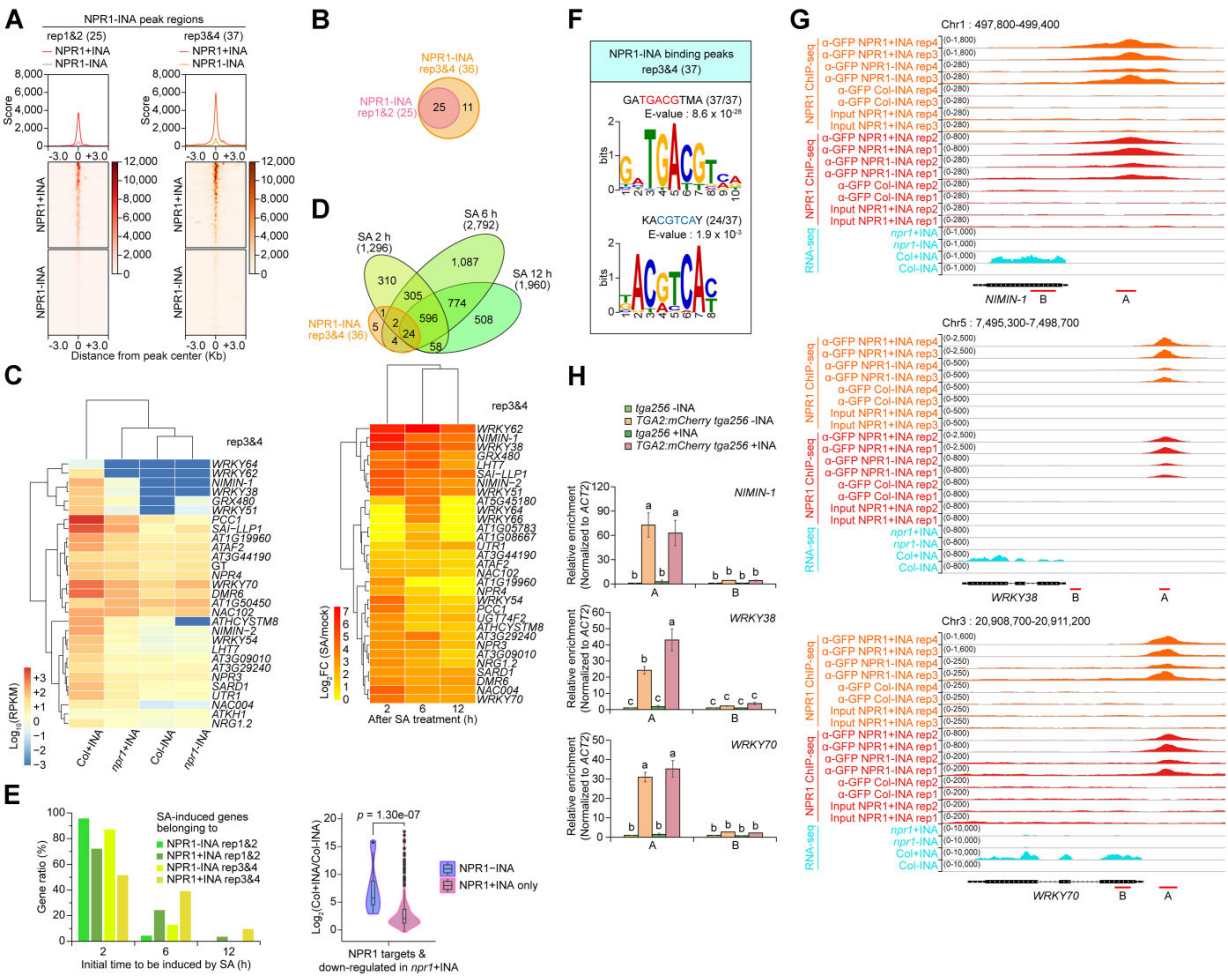
Genes targeted by NPR1 before SA signal show a tendency for more rapid and robust induction by SA. **(A)** Enrichment scores of NPR1:GFP in the absence (−INA) or presence (+INA) of INA within the regions enriched with NPR1:GFP in the absence of INA. Profile plots show the average scores of NPR1:GFP enrichment in regions from the 3 kb upstream to the 3 kb downstream of NPR1:GFP-peak centers. Heatmaps visualize enrichment scores corresponding to individual peaks. NPR1 targets identified from the rep1&2 or rep3&4 dataset were used for the analysis. See Figure 1A-B legend for more details. **(B)** Venn diagram showing the overlap between NPR1-target genes identified in the absence of INA (NPR1 pre-targets) from the two different ChIP-seq datasets consisting of two biological repeats each. Total numbers of annotated targets are indicated in parentheses. **(C)** Heatmap illustrating the expression levels of genes that were identified as NPR1 pre-targets from the rep3&4 dataset. Expression levels are presented as log_10_ values of RPKMs in WT (Col) and *npr1-1* mutant in the absence (−INA) or presence (+INA) of INA. Hierarchical clustering between genotype and/or treatment was performed based on similarity of gene expressions. **(D)** Expression of the NPR1 pre-targets identified from the rep3&4 dataset after 2, 6 and 12 h of SA treatment. RNA-seq data (29) obtained from BioProject database (ID: PRJNA224133) were used for analysis. The venn diagram illustrates the numbers of NPR1-target genes that are induced by 1 mM SA treatment at each time point. DEGs between SA and mock treatments were analyzed using two biological repeats including 4 technical runs each (log_2_FC ≥ 1, *P*-value < 0.05). The heatmap shows the expression level of each gene as log_2_ value of fold change (FC) between SA and mock treated WT. **(E)** The initial induction time after SA treatment and the induction fold changes by INA of the NPR1-target genes. Bar graph (left) illustrating the percentage of NPR1-target genes showing initial induction by SA. SA-induced genes presented in (D) were classified into four groups depending on their NPR1-targeting information provided by the rep1&2 and rep3&4 datasets. The genes in each group were further classified according to their initial induction time by SA, and the numbers of the classified genes were divided by the numbers of total genes of each group for gene-ratio calculation. Violin plot with included box plot (right) showing fold changes in NPR1-target expression before (−INA) and after (+INA) INA treatment in WT (Col) samples. From RNA-seq data (3), RPKMs in Col + INA were divided by RPKMs in Col − INA, and the log_2_ values of the calculated fold changes are presented. Among genes showing *NPR1*-dependent expression upon INA treatment, NPR1 pre-targets (NPR1 − INA) and only INA-dependent NPR1-target genes (NPR1 + INA only) were extracted and used for this analysis. The only INA-dependent NPR1-target genes were obtained by excluding the NPR1 pre-targets from the NPR1-target genes identified under INA treatment condition. *P*-value shown was calculated using two-tailed Mann-Whitney U-test. **(F)** DNA sequences enriched in NPR1 pre-targeting regions identified from the rep3&4 dataset. The results are displayed in descending order of E-value. The defined motif sequences are shown above E-values. Well-known motif sequences are in red for forward orientation or in blue for reverse orientation. Numbers of each motif occurrence are indicated in parentheses in comparison to the total numbers of input sequences. See Figure 3A legend for more details. **(G)** IGV snapshots of NPR1 ChIP-seq and RNA-seq data for representative genes displaying NPR1 pre-targeting. Red lines below gene models marked with A or B indicate NPR1-binding peaks or regions distant from the peaks, respectively. See Figure 3B legend for more details. **(H)** TGA2-targeting activity to NPR1 pre-targets. A and B regions indicated in **(G)** were amplified in ChIP-qPCR assays. See Figure 3C legend for more details.

We identified dozens of genes that are targeted by NPR1 without INA treatment and classified these genes as NPR1 − INA targets (Figure 1 and Supplementary Dataset S1). Most of these NPR1 − INA targets did not show *NPR1*-dependent expression in the absence of INA (Supplementary Figure S10A). Therefore, we investigated whether NPR1-targeting activity or expression of the NPR1 − INA targets might be changed by INA treatment. Upon INA treatment, NPR1 enrichment levels increased not only at the NPR1 − INA targets of the rep1&2 dataset but also at the targets of the rep3&4 dataset, which include all the rep1&2 NPR1 − INA targets (Figure 6A and B). Heatmaps for the NPR1 − INA targets showed that these genes are induced by INA in the WT, and we observed substantial expression differences between WT and *npr1* plants in the presence rather than in the absence of INA (Figure 6C and Supplementary Figure S10B). Thus, NPR1 targeting activity at the NPR1 − INA targets is reinforced by SA signaling, and further enriched NPR1 may lead to SA- and *NPR1*-dependent expression.

To better understand the biological importance of NPR1 pre-targeting, we investigated the RNA-expression dynamics of the NPR1 − INA targets identified from the rep3&4 dataset using published RNA-seq data (29). We found that 75% (27/36) of the NPR1 − INA targets were induced at 2 h after SA treatment, and this induction was maintained until 12 h after SA treatment (Figure 6D). In contrast, only 15% (157/1021) of the NPR1 + INA targets identified from the rep3&4 dataset were induced at 2 h after SA treatment (Supplementary Figure S10D). We obtained similar results when we investigated NPR1 targets identified from the rep1&2 dataset: 88% (22/25) or 29% (101/353) of the NPR1 − INA or NPR1 + INA targets, respectively, were induced at 2 h after SA treatment (Supplementary Figure S10C and D). When we compared initial induction times after SA treatment between the NPR1 − INA and NPR1 + INA targets among SA-induced genes, the NPR1 − INA targets tended to be induced more rapidly than the NPR1 + INA targets (Figure 6E). Furthermore, the induction fold of the NPR1 − INA targets was higher than that of the NPR1 targets identified only in the + INA condition (Figure 6E). Next, we performed a GO enrichment analysis on SA-induced NPR1 − INA targets and found that genes encoding DNA-binding factors known to be involved in SA-mediated signaling and defense responses are abundant. These include *SARD1*, *WRKYs* and *NACs* (Supplementary Figure S10E and Supplementary Dataset S11). These results indicate that NPR1 pre-targeting in the basal state results in more rapid and robust induction of the target genes preferentially encoding transcriptional regulators during SA-triggered immunity.

We then asked which transcription factors mediate NPR1 targeting in the absence of INA. Motif analyses using NPR1 − INA target-site sequences revealed the TGACG motif as the sole transcription factor-binding site (Figure 6F and Supplementary Figure S10F). We then selected three of the NPR1 − INA targets, *NIM1-INTERACTING1* (*NIMIN-1*), *WRKY38*, and *WRKY70*, to test TGA2 enrichment in their NPR1-targeting regions containing the TGACG motif, as these genes exhibited increased NPR1 targeting by INA treatment and INA- as well as *NPR1*-dependent expression (Figure 6G). Within the NPR1-targeting regions of these genes, TGA2:mCherry showed an INA-independent targeting activity (Figure 6H). NPR1 targeting activity to the regions targeted by TGA2:mCherry was reconfirmed by ChIP-qPCR assays, both before and after INA treatment (Supplementary Figure S10G). Therefore, NPR1 targeting in the basal state is also mediated by TGA transcription factors.

## Discussion

**Figure 7.**
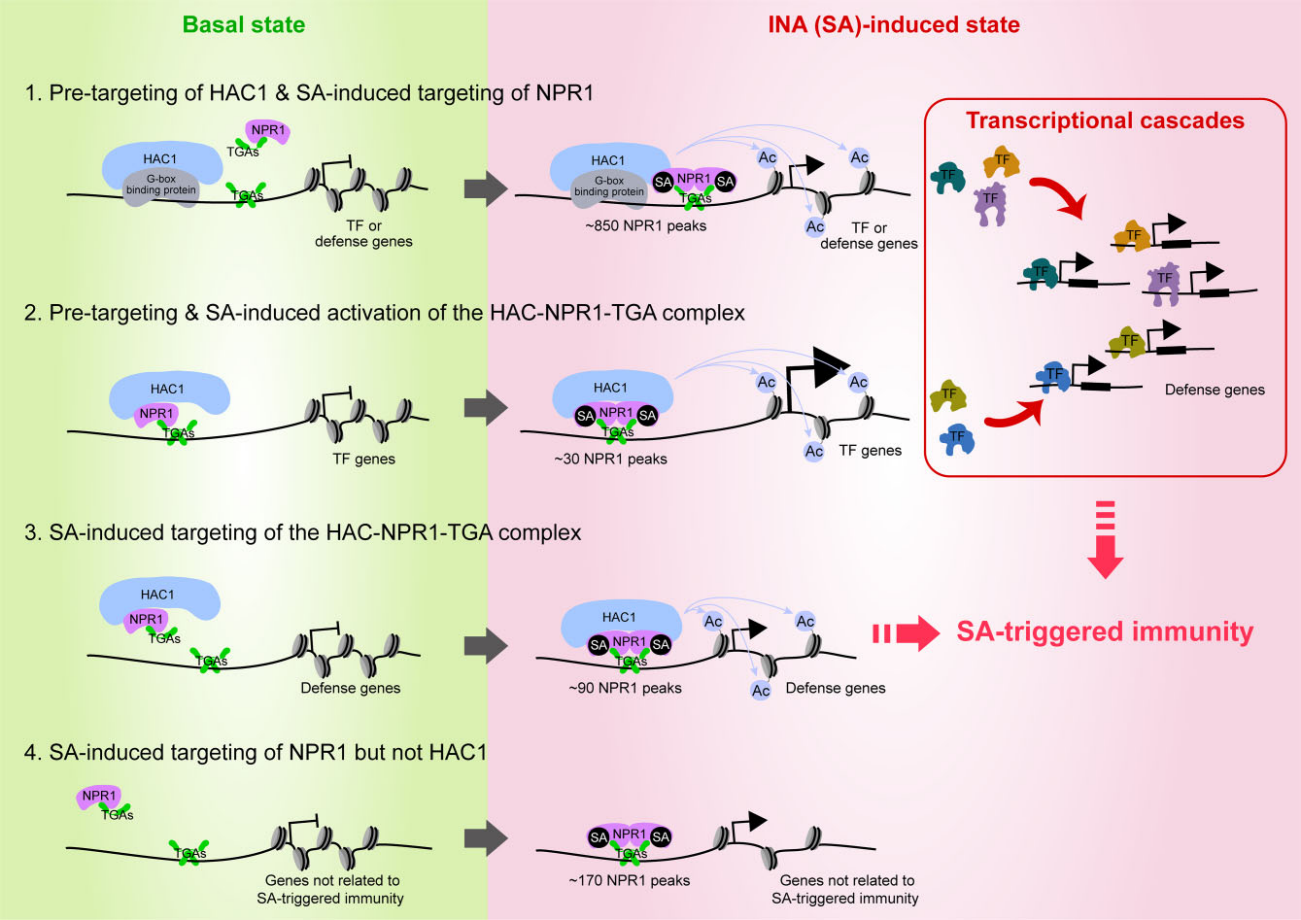
A model for INA (SA)-induced transcriptional reprogramming through genome-wide targeting of NPR1 and subsequent transcriptional cascades. Based on the targeting activities of NPR1 and HAC1 onto chromatin before and after INA treatment, NPR1 targets might be classified into four groups. The first group of the NPR1 targets, which includes the majority of the NPR1 targets, might be pre-targeted by HAC1 in the basal state probably through transcription factor(s) binding to the G-box motif. INA (SA)-dependent NPR1 targeting to these HAC1 pre-targets causes an increase in H3Ac level and transcriptional activation. Increased expression of transcription factor (TF)-encoding genes, which constitute the largest subgroup within the first group, triggers transcriptional cascades and subsequent activation of thousands of defense genes. Genes involved in defense responses other than transcriptional regulation are also included in the first group. In the second group of the NPR1 targets, the HAC-NPR1-TGA complex might be pre-targeted in the basal state, while NPR1 enrichment and H3Ac levels increase upon INA (SA) signaling. These NPR1 pre-targets, which mostly encode TFs, are rapidly and robustly induced upon INA (SA) signaling and allow for fast and extensive transcriptional cascades. In the third group of the NPR1 targets, which generally constitutes defense genes not encoding TFs, the HAC-NPR1-TGA complex might be targeted in an INA (SA)-dependent manner and causes transcriptional activation along with increased H3Ac levels. In the fourth group of the NPR1 targets, which is composed of genes not in the SA-triggered immunity pathway, NPR1 is targeted independently of HAC1 upon INA (SA) signaling and results in the transcriptional activation of subset of the target genes. In all cases, TGAs might target the majority of the NPR1 targets by binding to the TGA or G-box motifs independently of INA (SA) signaling.

NPR1 plays a crucial role in SA-induced transcriptional reprogramming which leads to SA-triggered immunity in plants. In this study, we uncovered INA-specific targets of NPR1 including co-targets shared by NPR1 and HAC1 at genomic level. Based on our findings and analyses, we present a comprehensive model for the transcriptional reprogramming triggered by INA (SA)-dependent NPR1 targeting and co-targeting of NPR1 and HAC1 (Figure 7).

Our genome-wide study revealed that NPR1 targets to the genome mostly in an INA-dependent manner and primarily activates genes encoding DNA-binding factors through its direct targeting. The proportion of direct NPR1 targets accounted for only 3% (116/3675 based on the rep1&2 dataset) or 8% (278/3675 based on the rep3&4 dataset) of the *NPR1*-dependently expressed genes. Among these *NPR1*-dependently expressed NPR1 targets, genes encoding DNA-binding factors formed the largest group. On the other hand, most *NPR1*-dependently expressed defense-related genes were not directly targeted by NPR1. These results suggest that NPR1 elicits transcriptional cascades upon SA perception during genome-wide transcriptional reprogramming that confers host plant immunity. Among various families of transcription factor genes directly targeted and regulated by NPR1, the *WRKY* family was the most abundant. The importance and dominance of WRKYs in SA-triggered transcriptional reprogramming has been studied (45–48). In addition to *WRKYs*, we identified genes encoding a variety of other types of transcription factors. Thus, diverse families of transcription factors may mediate the SA-induced transcriptional cascades initiated by NPR1 targeting.

Besides triggering transcriptional cascades, NPR1 directly activates various biological processes upon SA perception. For example, our identification of receptor-, NADPH oxidase-, ABA transporter-, protein kinase- and diverse ETI regulator-encoding genes as direct NPR1 activation targets demonstrates a broad, direct role of NPR1 in SA-triggered immunity. Hence, the master regulatory role of NPR1 seems to be executed through directly activating key regulatory components of SA-dependent immunity as well as triggering transcriptional cascades.

Balancing the amplitude of immune response is critical for optimal plant fitness. The finding that NPR1 also targets and activates negative regulators of SA-triggered immunity upon SA signaling suggests a role of NPR1 in balancing SA-triggered immunity. WRKYs positively or negatively regulate SA-triggered immunity (49–51). Our study showed that WRKYs that negatively regulate SA-triggered immunity, such as WRKY18, WRKY38, WRKY40 and WRKY62, are encoded by a group of genes that are directly targeted by NPR1 and activated in INA- and *NPR1*-dependent manners. Another member of this group was NIMIN-1, which inhibits NPR1 during SA-induced *PR1* induction by forming a complex with NPR1 (52). NPR1 homologs, such as NPR3 and NPR4, that were also found in this group, are SA receptors that cause NPR1 degradation (53) and act as transcriptional repressors, unlike NPR1 (10). *DOWNY MILDEW RESISTANT6* (*DMR6*) was another NPR1 target activated in an *NPR1*-dependent manner upon INA treatment. *DMR6* encodes an SA 5-hydroxylase, which controls SA homeostasis by catalyzing the hydroxylation of SA, and acts as a negative regulator of SA-induced immunity (54). Thus, NPR1 not only activated positive regulators but also negative regulators of SA-triggered immunity. Without the opposing function of these negative regulators, NPR1 activity might generate excessive or prolonged immune responses that could be deleterious to plant fitness and create vulnerability to other stresses. Therefore, we suggest that NPR1 might balance immune responses by directly activating positive and negative defense pathways, and this balancing might enhance plant fitness and survival.

Our study also revealed that NPR1 is directly involved in phytohormone crosstalk. For example, NPR1 targeted to and activated *WRKY46*, *WRKY54*, and *WRKY70*, which are involved in brassinosteroid (BR) biosynthesis (55). BR enhances SA-triggered immunity by inhibiting the BRASSINOSTEROID-INSENSITIVE2 (BIN2)-mediated phosphorylation and destabilization of clade I TGAs (56). Thus, NPR1 might reinforce SA-triggered immunity by increasing BR biosynthesis. Genes encoding several WRKYs involved in JA and ethylene (ET) signaling were also among the genes directly targeted and activated by NPR1. SA and JA/ET signaling pathways have been described to have an antagonistic relationship (1,57). On the contrary, it has also been reported that SA and JA/ET synergistically regulate SA-responsive genes (58,59) or programmed cell death during ETI (60) and that key components in JA/ET pathways positively regulate SA-responsive genes during immunity (61,62). Our study showed that NPR1 directly activates genes encoding ethylene response factors (ERFs) that are major transcriptional regulators of ET-responsive genes. Thus, we propose that NPR1 coordinates complex phytohormone signaling networks during SA-triggered transcriptional reprogramming, probably to optimize SA-triggered immunity.

This study uncovered that NPR1 and HAC1 co-targeting at the genome level is involved in *NPR1*- and *HAC1/5*-dependent INA-induced transcriptional reprogramming. Our ChIP-seq analyses revealed several hundred NPR1 and HAC1 co-targets. At these co-targeting loci, INA-induced H3Ac levels increased in *NPR1*- and *HAC1/5*-dependent manners, and a subset of the co-targets also showed *NPR1*- and *HAC1/5*-dependent expression upon INA treatment. These results are consistent with our previous study that reported HAC1/5 as epigenetic factors recruited by the NPR1-TGA complex to confer transcriptional coactivator function to NPR1 (3). Our current study furthers our understanding of the cooperative roles of NPR1 and HAC1/5 at the genome level. Together, our studies demonstrate that HAC1 is a bona fide epigenetic partner of NPR1 acting at several hundred co-targets upon INA signaling to promote the expression of at least dozens of co-targets. A recent structure-based study proposed an enhanceosome model in which an SA-induced structural change of NPR1 facilitates recruitment of an unknown transcriptional regulator(s) for gene activation (11), consistent with the idea that HAC1 cooperates with NPR1 to regulate SA-induced transcriptional reprogramming. However, it remains unknown how NPR1 induces transcription at *NPR1*-dependent but *HAC1/5*-independent loci during SA-triggered transcriptional reprogramming. Thus, it would be of interest to find new epigenetic partners of NPR1.

We also found that NPR1 and HAC1 co-targeting as well as NPR1 targeting are principally mediated by TGA transcription factors. Consistently, the TGACG sequence was the DNA motif most abundantly found within NPR1 targeting regions, both in the absence or presence of INA. In addition, the TGACG motif was the most abundant even in the co-targeting regions of NPR1 and HAC1 identified upon INA treatment. Further, TGA2, a representative TGA that directly interacts with NPR1 (15,16), bound independently of INA to the NPR1 targets and the NPR1 and HAC1 co-targets containing the TGACG motif. Unexpectedly, TGA2 also bound to NPR1 targets containing the G-box but not the TGACG motif. With this regard, it would be worthy to note that a previous study showed the binding of recombinant TGA1a and TGA1b proteins to oligonucleotides containing the G-box motif (63). This suggests that TGA2 may also directly bind to the G-box motif and recruit NPR1 or the HAC-NPR1 complex to its target loci, highlighting the crucial role of TGAs in the genome-wide targeting of NPR1 and the co-targeting of NPR1 and HAC1. However, it is still possible that TGA2 may indirectly bind to the G-box within NPR1 targets via another transcription factor(s). For this reason, it would be interesting to find another transcription factor(s) mediating the recruitment of the HAC-NPR1-TGA (3) or NPR1-TGA complex to the NPR1 targets containing non-TGACG motifs, especially the G-box.

Dozens of genes were already targeted by NPR1 independently of INA treatment, and these NPR1 pre-targets were more rapidly and robustly induced by SA compared to INA-dependent NPR1 targets. NPR1 targeting of genes in the basal state probably makes the target chromatin more accessible, allowing drastically increased NPR1 recruitment upon SA signaling. Alternatively, SA binding to pre-targeted NPR1 may lead to an NPR1 conformation efficient for transcriptional activation on site. Genes involved in transcriptional regulation, such as *WRKYs*, *NACs*, *NIMINs*, *NPRs* and *SARD1*, were abundantly represented among the NPR1 pre-targets. This suggests that NPR1 pre-targeting is likely to initiate rapid and extensive transcriptional cascades, accelerating SA-triggered transcriptional reprogramming.

In summary, our genome-wide study revealed that the primary role of NPR1 is to directly activate genes encoding DNA-binding factors through INA (SA)-dependent targeting. In addition to transcription factor-encoding genes, NPR1 directly activated genes involved in various biological processes required for SA-triggered immunity. Furthermore, NPR1 directly activated positive and negative regulators of SA-triggered immunity and genes involved in phytohormone crosstalk possibly to balance defense responses. A subset of NPR1 targets required HAC1/5 as epigenetic partners, and TGA transcription factors played a major role in recruiting NPR1 and the NPR1-HAC1 complex to genome-wide targets. Furthermore, NPR1 bound to some targets in the basal state, and this occupancy allowed for more rapid and robust target induction upon SA signaling, particularly the transcriptional activation of genes encoding transcriptional regulators. Thus, our study uncovers genome-wide targeting activities of NPR1 and HAC1, their roles in INA (SA)-induced transcriptional reprogramming, and the cooperativity between NPR1, HAC1, and TGAs in conferring immunity to Arabidopsis.

## Supplementary Material

gkae019_Supplemental_Files

## Data Availability

The ChIP-seq data have been deposited in the Gene Expression Omnibus (GEO; https://www.ncbi.nlm.nih.gov/geo/) under the SuperSeries accession number GSE211047. All other data are available from the authors upon reasonable request.
