# Supplementary material for: Genomic overview of INA-induced NPR1 targeting and transcriptional cascades in Arabidopsis
Source: Nucleic Acids Res. 2024 Jan 23;52(7):3572–88. doi: 10.1093/nar/gkae019 (PMC11039990; doi:10.1093/nar/gkae019)
Supplement: gkae019_Supplemental_Files [file gkae019_supplemental_files.zip › Yun_Supplementary Data_revised.pdf]

SUPPLEMENTARY DATA

Genomic overview of INA-induced NPR1 targeting and transcriptional cascades in Arabidopsis

Se-Hun Yun[1,2], Irfan Ullah Khan[1,2], Bosl Noh[3,]* & Yoo-Sun Noh[1,2,]*

[1]School of Biological Sciences, Seoul National University, Seoul 08826, Korea, [2]Research Center for Plant Plasticity, Seoul National University, Seoul 08826, Korea, [3]Research Institute of Basic Sciences, Seoul National University, Seoul 08826, Korea

*To whom correspondence should be addressed. Tel: +82 2 880 6674; Fax: +82 2 882 4421; Email: ysnoh@snu.ac.kr
Correspondence may also be addressed to: Bosl Noh. Tel: +82 2 871 6675; Fax: +82 2 882 4421; Email: bnoh2003@gmail.com

- Supplementary Figures S1 to S10

- Supplementary Tables S1 to S3

Supplementary Figure S1

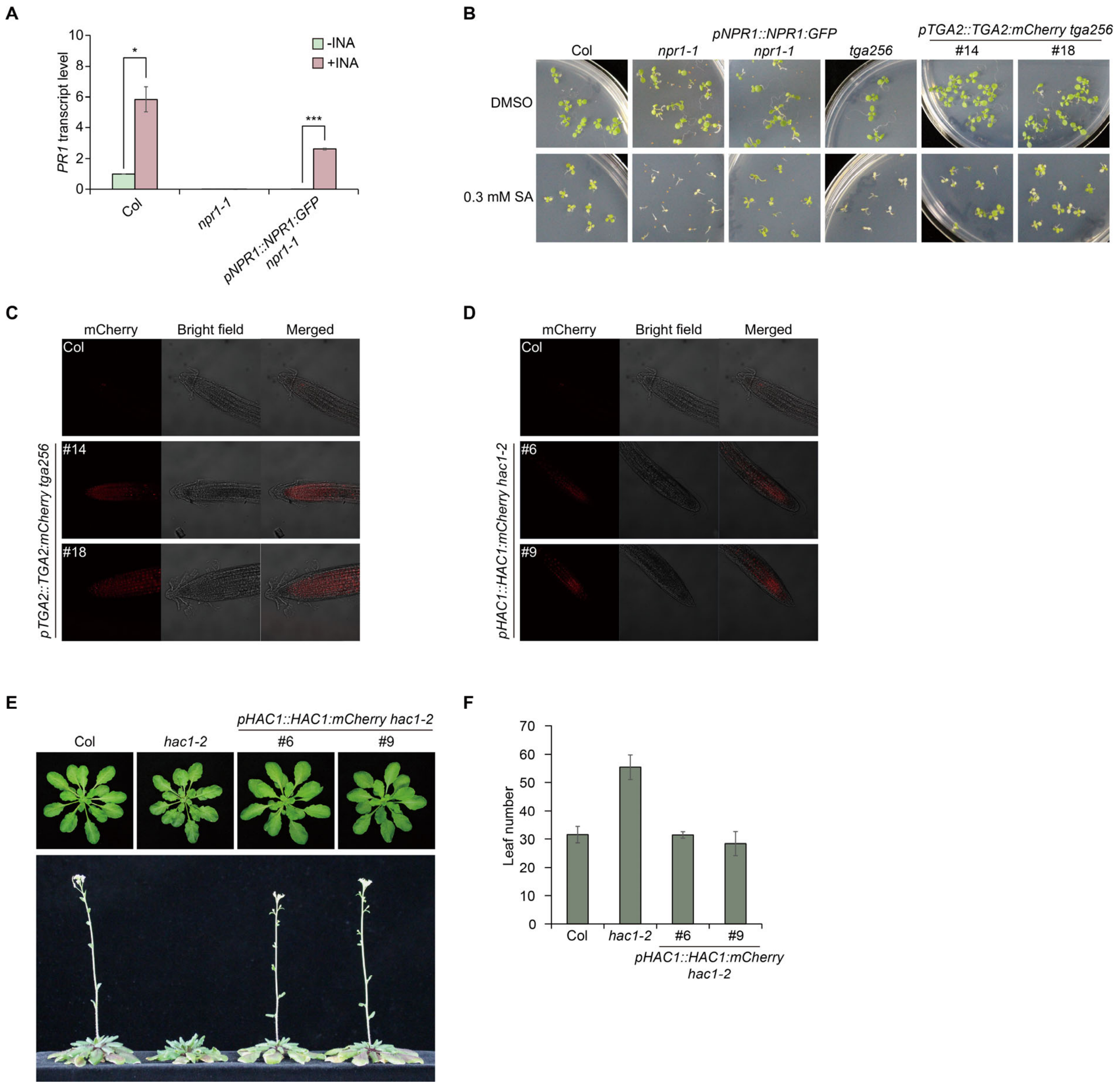

Supplementary Figure S1. Functional complementation assays of *pNPR1::NPR1:GFP*, *pTGA2::TGA2:mCherry*, and *pHAC1::HAC1:mCherry* that were introduced into the *npr1-1*, *tga2 tga5 tga6* (*tga256*), and *hac1-2* mutant, respectively. (**A**) RT-qPCR analyses of *PR1* transcript levels in wild-type (Col), *npr1-1* mutants, and *pNPR1::NPR1:GFP npr1-1* transgenic plants treated with 0.3 mM INA (+INA) or not (−INA). The relative transcript levels were calculated by setting the transcript level of Col−INA to 1 after normalization to *ubiquitin10* (*UBQ10*). Means ± SE of 3 biological replicates are shown. Asterisks indicate statistically significant differences between −INA and +INA conditions (**p*-value < 0.05 and ****p*-value < 0.001 in a Student's *t*-test). All plants were grown on the soil under 8-hour (h) light/16-h dark (8L/16D) photoperiod. (**B**) Representative images of Col, *npr1-1*, *pNPR1::NPR1:GFP npr1-1*, *tga256*, and *pTGA2::TGA2::mCherry tga256* that were grown on the Murashige and Skoog (MS) medium supplemented with either 0.05% dimethyl sulfoxide (DMSO) as a control or 0.3 mM salicylic acid (SA) pre-mixed with DMSO. All seedlings were incubated under 16L/8D photoperiod for 10 days. #18 line of *pTGA2::TGA2::mCherry tga256* plants was used for all the rest of experiments in this study. (**C-D**) Confocal microscopy images of the root tips of Col and two independent transgenic-line plants of *pTGA2::TGA2:mCherry tga256* (**C**) or *pHAC1::HAC1:mCherry hac1-2* (**D**). (**E**) Representative images of Col, *hac1-2*, and the two independent transgenic-line plants of *pHAC1::HAC1:mCherry hac1-2* grown on the soil under 12L/12D photoperiod. (**F**) The flowering time of Col, *hac1-2*, and the two independent *pHAC1::HAC1:mCherry hac1-2* transgenic-line plants grown under 12L/12D photoperiod. Means ± SD are shown (n=7). #9 line of *pHAC1::HAC1:mCherry hac1-2* plants was used for all the rest of experiments in this study.

Supplementary Figure S2

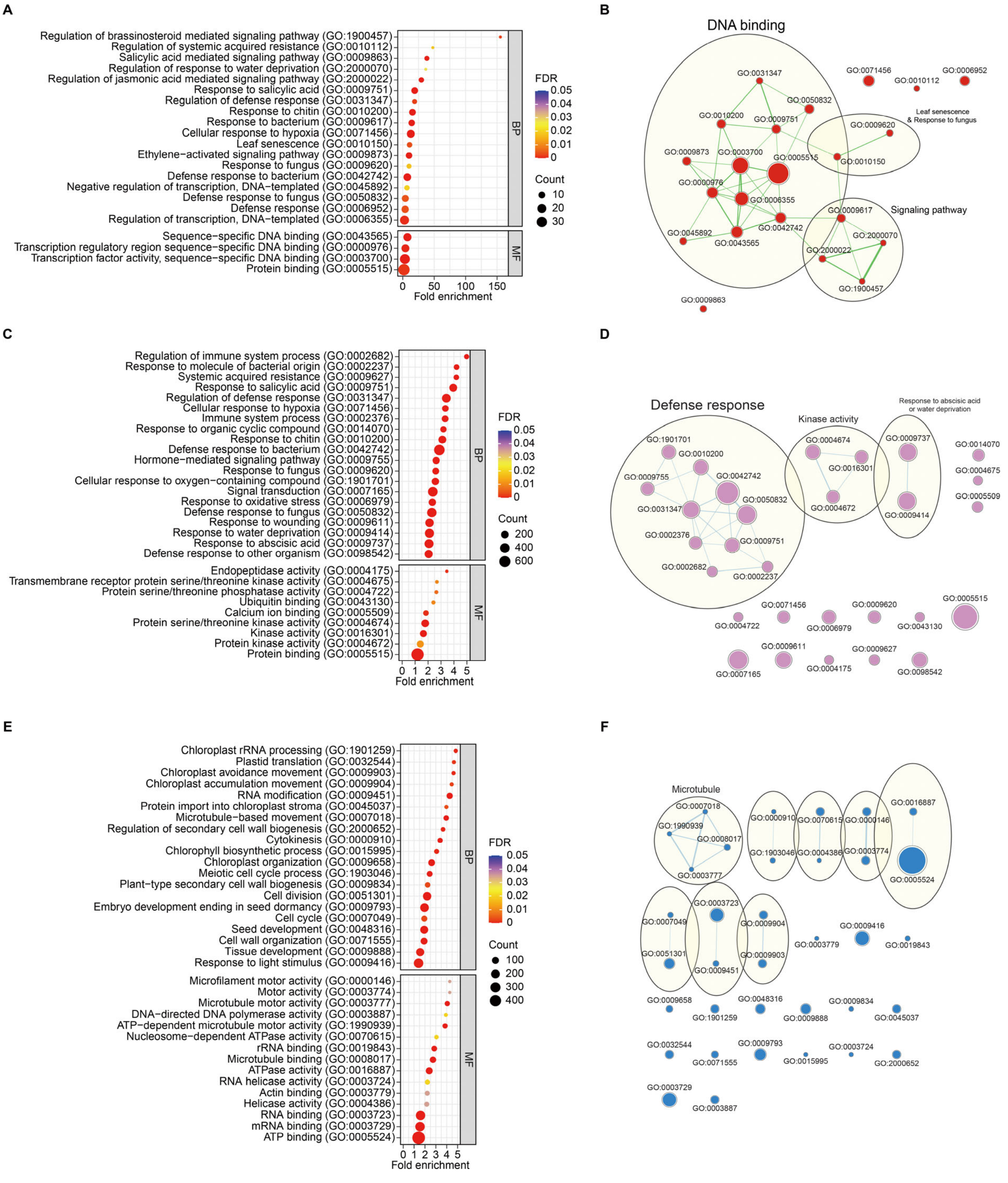

Supplementary Figure S2. Ontology of *NPR1*-regulated genes that are either directly targeted by NPR1 or not upon INA treatment. (**A**, **C**, **E**) GO terms enriched among genes showing *NPR1*-dependent regulation and either directly targeted by NPR1 or not upon INA treatment. The enriched GO terms were selected with cutoff of FDR < 0.05. See Figure 2C legend for more details. (**A**) presents GO terms enriched among genes that are directly targeted and activated by NPR1 upon INA treatment. NPR1 targets within the rep1&2 dataset of ChIP-seq were analyzed. (**C** and **E**) present GO terms enriched among genes that are NPR1-dependently activated (**C**) or repressed (**E**) but not directly targeted by NPR1 upon INA treatment. NPR1 targets within the rep3&4 dataset of ChIP-seq were excluded from the total genes showing *NPR1*-dependent activation (for C) or repression (for E) in the presence of INA. Top 20 results in ascending order of FDR were chosen and displayed for biological process (BP). (**B**, **D**, **F**) Enrichment map visualizing the networks and clusters of gene sets obtained from the GO analysis in (A), (C), and (E), respectively. The gene sets were selected with Q-value < 0.05, and the edge cutoff meaning a similarity between a pair of gene sets was 0.25 with 0.15 of Jaccard and overlap combined constant. See Figure 2D legend for more explanations.

Supplementary Figure S3

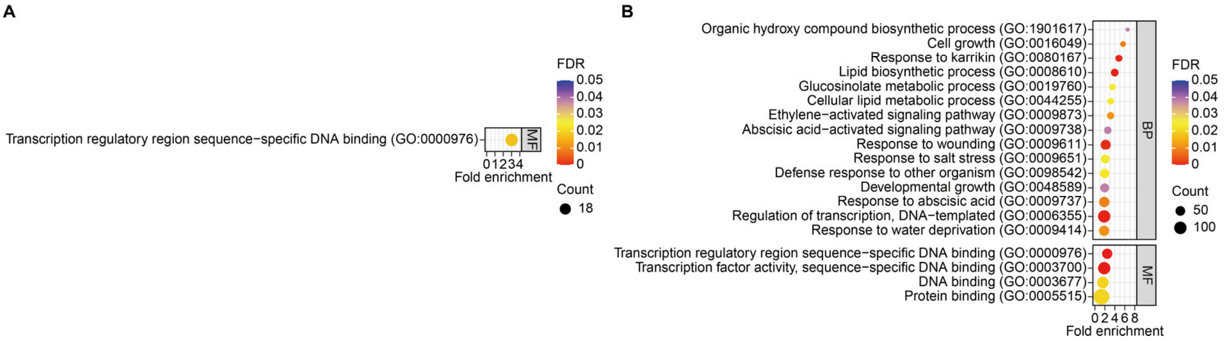

Supplementary Figure S3. Ontology of direct NPR1 target genes that are not downregulated in *npr1* mutant upon INA treatment within the rep1&2 (**A**) or the rep3&4 (**B**) dataset of ChIP-seq. The enriched GO terms were selected with cutoff of FDR < 0.05. See Figure 2C legend for more details.

Supplementary Figure S4

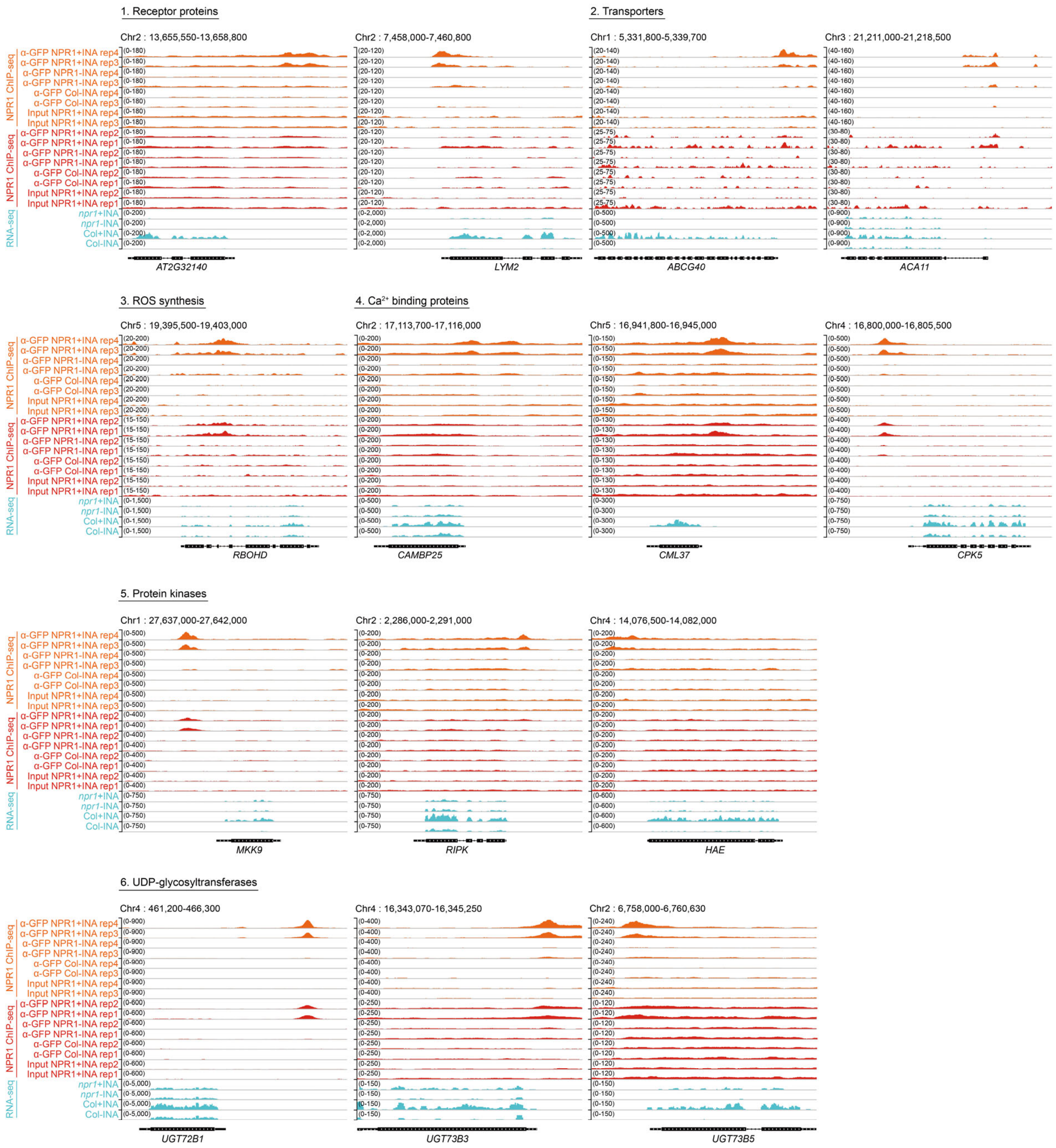

Supplementary Figure S4. IGV snapshots of the functionally classified direct NPR1-targets showing *NPR1*-dependent expression in the presence of INA. Integrative Genomics Viewer (IGV) snapshots of NPR1 ChIP-seq and RNA-seq data for representative functionally classified genes that are targeted and activated by NPR1 upon INA treatment. Data scales are indicated in parentheses on the right side of y-axes. Chromosome (Chr) numbers and genomic regions are shown at the top of images.

Supplementary Figure S5

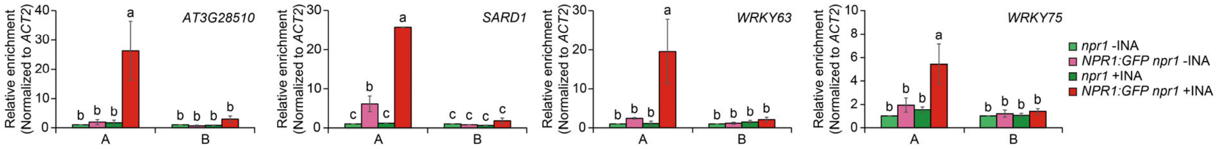

Supplementary Figure S5. NPR1-targeting activity to genomic regions where NPR1 peaks containing the TGACG sequences (TGA-binding motifs) are located. NPR1:GFP enrichment was determined without (−INA) or with (+INA) INA treatment by ChIP-qPCR. Genomic regions (A and B) that were tested are shown in Figure 3B. To calculate relative enrichments, the values of *npr1*−INA were set to 1 after normalization by input and *actin2* (*ACT2*). See Figure 3C legend for more details.

Supplementary Figure S6

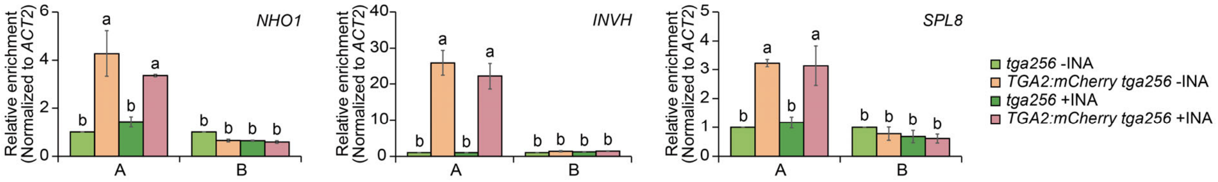

Supplementary Figure S6. TGA2-targeting activity to the NPR1-targeting regions containing the CACGTG sequences (G-box motifs) but not the TGACG sequences (TGA-binding motifs). TGA2:mCherry enrichment was determined without (–INA) or with (+INA) INA treatment by ChIP-qPCR. Tested regions (A and B) are described in Figure 3B. See Figure 3C legend for more details.

Supplementary Figure S7

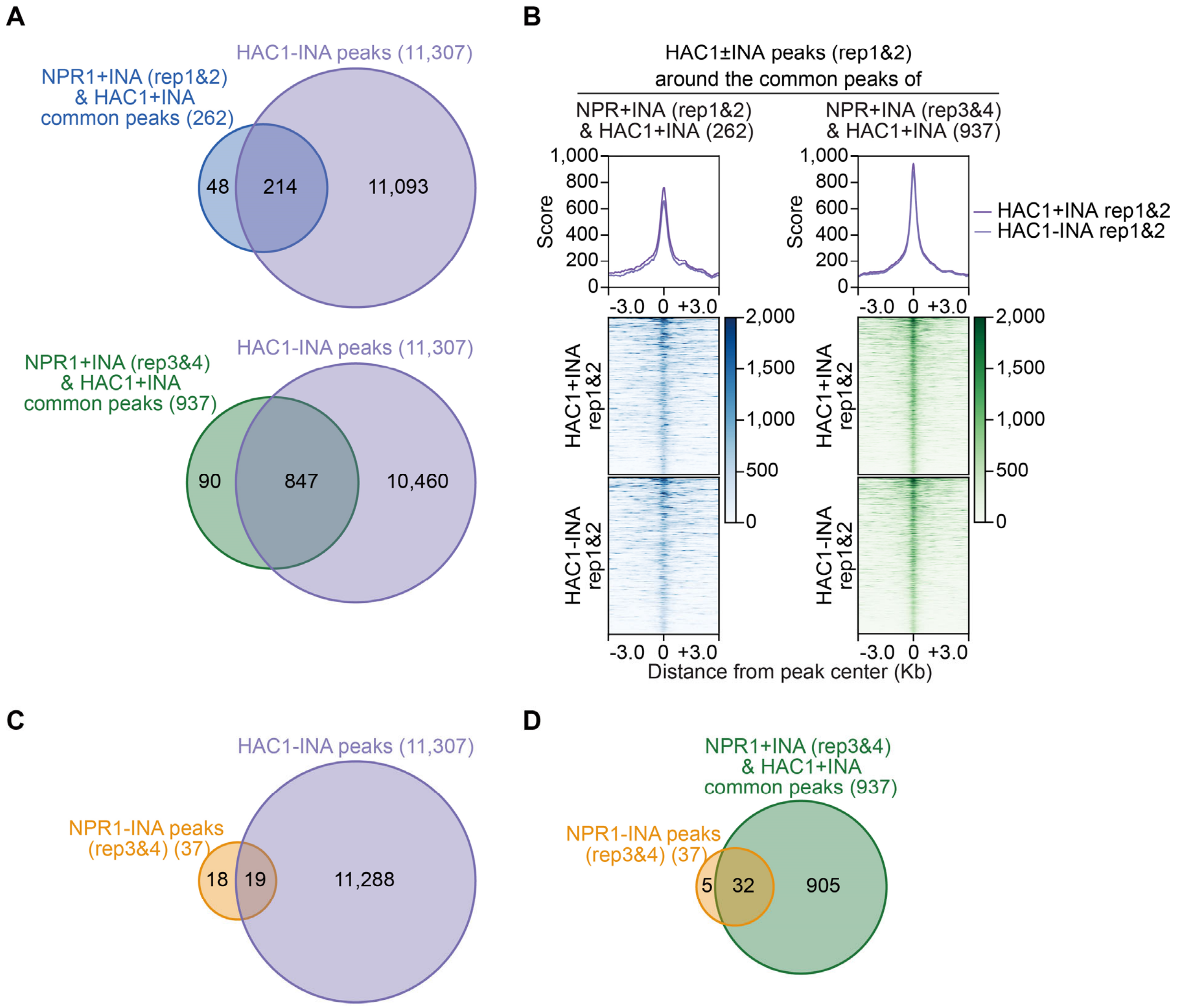

Supplementary Figure S7. Overlaps of NPR1 and/or HAC1 peaks in the absence (−INA) or presence (+INA) of INA. (**A, C, D**) Venn diagrams showing the overlaps between HAC1−INA peaks and the common peaks of NPR1+INA and HAC1+INA (NPR1+INA & HAC1+INA) (**A**), NPR1−INA and HAC1−INA peaks (**C**), or NPR1−INA peaks and NPR1+INA & HAC1+INA common peaks (**D**). The total numbers of peaks are indicated in parentheses next to their respective names. (**B**) Enrichment scores of HAC1:mCherry under −INA or +INA conditions around the NPR1+INA & HAC1+INA common peaks. Profile plots show average scores of HAC1:mCherry enrichment in the regions surrounding the common peaks. Heatmaps show the corresponding enrichment scores for individual HAC1 peaks. See Figure 4A and 4D legends for more details.

Supplementary Figure S8

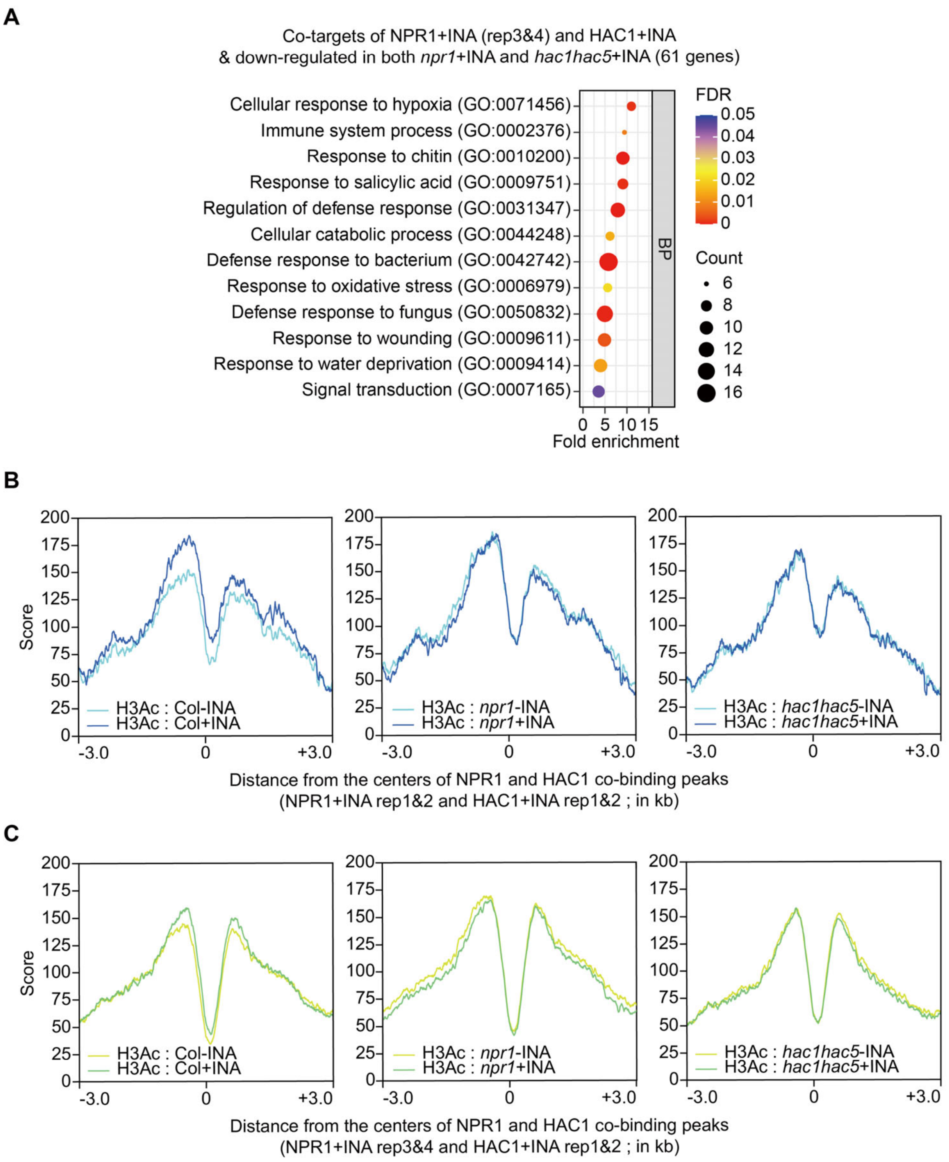

Supplementary Figure S8. Ontology and histone H3 acetylation (H3Ac) levels of NPR1 and HAC1 co-targets. (**A**) GO terms enriched among genes directly co-targeted and co-activated by NPR1 and HAC1 in the presence of INA. Co-targets identified from the rep3&4 dataset of NPR1:GFP ChIP-seq were analyzed. The enriched GO terms were selected with cutoff of FDR < 0.05. See Figure 2C legend for more details. (**B-C**) Profile plots showing H3Ac levels before and after INA treatment in WT (Col), *npr1-1*, and *hac1-2 hac5-2*. The H3Ac profiles were analyzed using the H3Ac ChIP-seq data previously reported (3). Divided into 5 bins, genomic regions were scanned from the 3 kb upstream to the 3 kb downstream of the centers of ChIP-seq peaks that show co-targeting activities of NPR1 and HAC1 in the presence of INA (see Figure 4D). Co-targeting sites identified from the rep1&2 (**B**) or the rep3&4 (**C**) of NPR1:GFP ChIP-seq dataset were analyzed.

Supplementary Figure S9

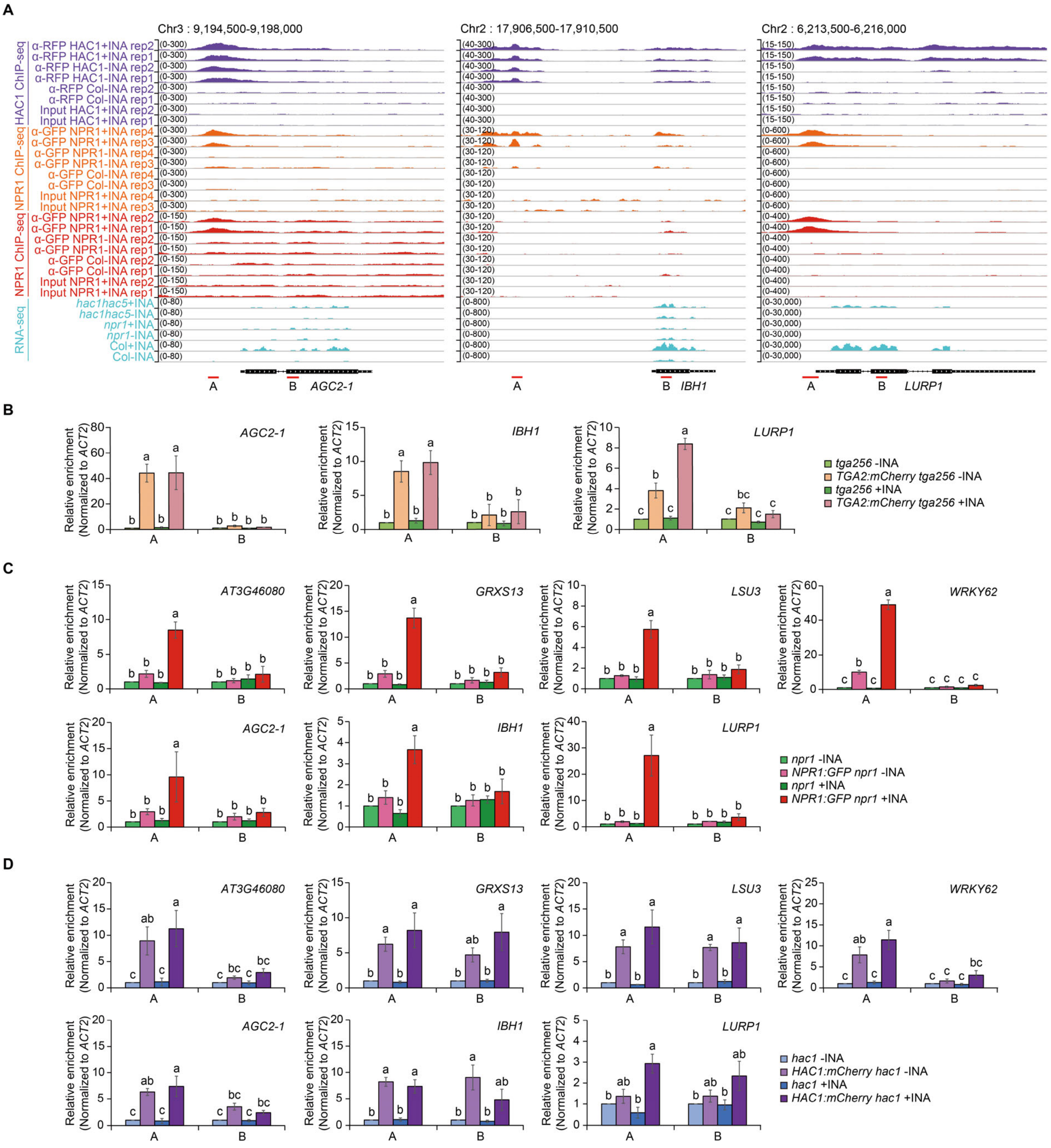

Supplementary Figure S9. TGA2 targeting to regions co-targeted by NPR1 and HAC1. (**A**) IGV snapshots of HAC1 ChIP-seq, NPR1 ChIP-seq, and RNA-seq data for three representative co-targets of NPR1 and HAC1. The representative co-targets are the genes that are co-activated by both *NPR1*- and *HAC1 HAC5*-dependent manners and contain the TGACG motifs within the common peaks of NPR1 and HAC1. See Figure 5B and 3B legends for more details. (**B**) TGA2-targeting activity to the common peaks containing the TGACG motif (regions A) or distant regions (regions B) presented in (A) in the presence (+INA) or absence (−INA) of INA. See Figure 5C and 3C legends for more details. (**C-D**) NPR1- or HAC1-targeting activity at regions containing the common peaks of NPR1 and HAC1 and the TGACG motifs. Both NPR1:GFP (**C**) and HAC1:mCherry enrichments (**D**) under −INA and +INA conditions were determined by ChIP-qPCR. Genomic regions (A and B), shown in Figure 5B and Supplementary Figure S9A, were tested. To calculate relative enrichments, the values of *npr1*−INA or *hac1*−INA were set to 1 after normalization by input and *actin2* (*ACT2*). See Figure 3C legend for more details.

Supplementary Figure S10

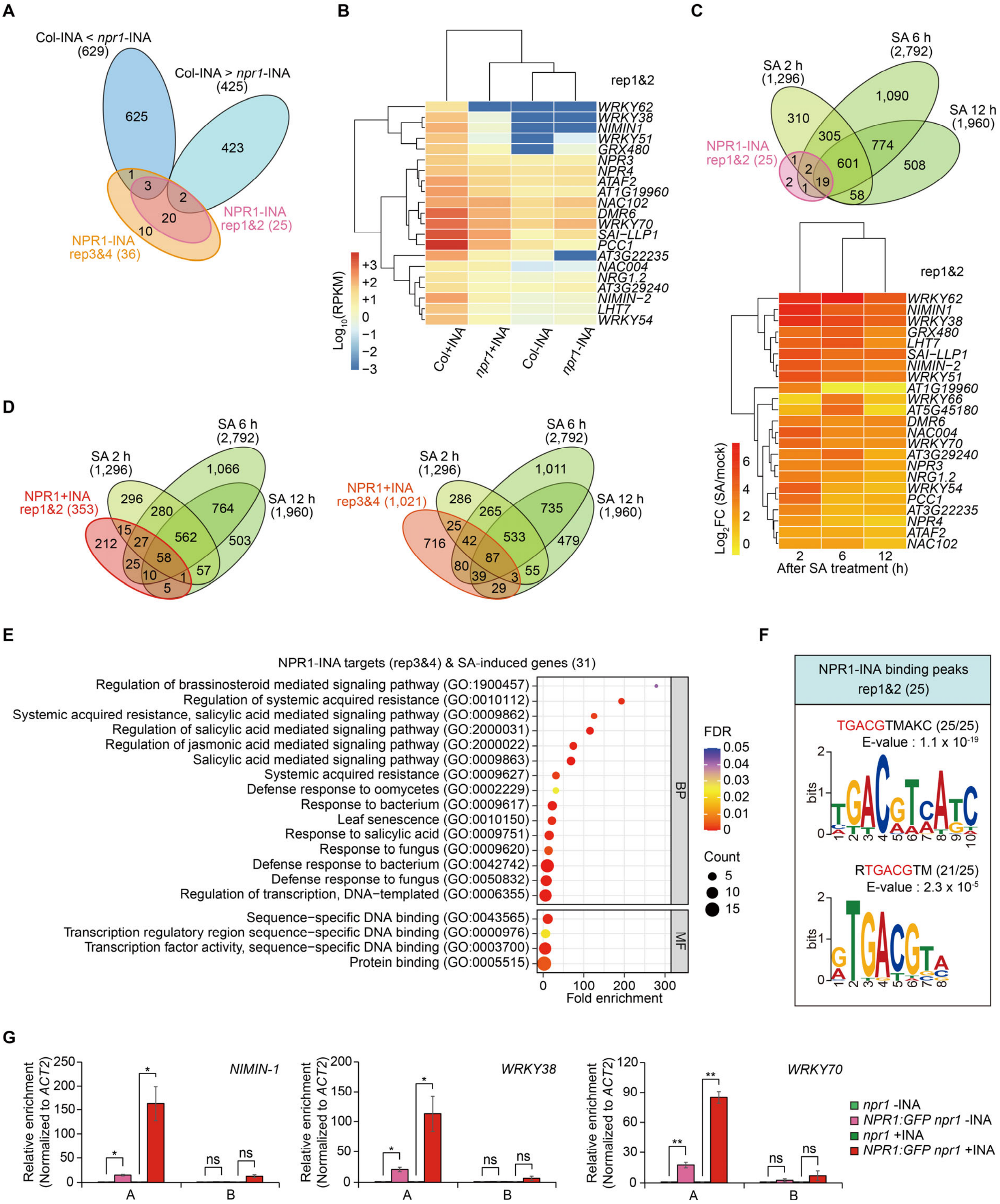

Supplementary Figure S10. INA-independent NPR1 targets show a tendency for rapid induction by SA and are enriched with genes encoding DNA-binding factors. (**A**) Venn diagram illustrating the overlaps between INA-independent NPR1 targets and genes showing *NPR1*-dependent expression in the absence of INA. (**B**) Heatmaps illustrating the expression levels of INA-independent NPR1 targets identified from the rep1&2 dataset. See Figure 6C for more details. (**C**) Expression of the INA-independent NPR1-target genes identified from the rep1&2 dataset after 2, 6, and 12 h of SA treatment. See Figure 6D legend for more details. (**D**) Venn diagrams illustrating the numbers of genes showing INA-dependent NPR1 targeting and SA-induced expression over time. NPR1 targets identified from the rep1&2 or the rep3&4 dataset were analyzed separately. (**E**) GO terms enriched among INA-independent NPR1 targets induced by SA. NPR1−INA peaks identified from the rep3&4 dataset were used here. The enriched GO terms were selected with cutoff of FDR < 0.05. See Figure 2C legend for more details. (**F**) DNA sequences enriched in INA-independent NPR1-targeting regions identified from the rep1&2 dataset. The results are displayed in descending order of E-value. The defined motif sequences are shown above E-values. Well-known motif sequences are in red. Numbers of each motif occurrence are indicated in parentheses in comparison to the total numbers of input sequences. See Figure 3A legend for more details. (**G**) NPR1-targeting activity to regions where INA-independent NPR1 peaks are located. NPR1:GFP enrichment under −INA and +INA conditions was determined by ChIP-qPCR. Genomic regions (A and B) shown in Figure 6G were tested. To calculate relative enrichments, the values of *npr1*−INA and *npr1*+INA were set to 1 after normalization by input and *actin2* (*ACT2*). Means ± SE of three biological replicates are shown. Asterisks indicate statistically significant differences between *npr1-1* and *pNPR1::NPR1:GFP npr1-1* (**p*-value < 0.05 and ***p*-value < 0.01 in a Student's *t*-test). 'ns' means a statistically no significant difference in a Student's *t*-test.

Supplementary Table S1. Primers used for plasmid constructions.

Name	Sequence
HAC1 promoter-F	5'- CACCGATTTGGGAAAACCTGAATTCATTCGCT -3'
HAC1-R (w/o stop)	5'- ACCTGAGCCCCCAGCGACTTCTGCAGCTC -3'
NotI-TGA2 promoter-F	5'-CAAGGCGGCCGCTAATGAGTTAAGAATAGAGAATG-3'
NdeI-TGA2 promoter-R	5'-CTTGCATATGATTACTTTCTCACCACTTTTCTGTAC-3'
NdeI-TGA2-ORF-F	5'-CACCCATATGGCTGTACCAGTCCGAGAAC-3'
TGA2-ORF-R (w/o stop)	5'-CTCTCTGGGTCGAGCAGCCATAAGG-3'
AscI-mCherry-F	5'-GCGGGCGCGCCATGGTGAGCAAGGGCGAG-3'
PacI-mCherry-stop-R	5'-GGATTAATTAATCACTTGTACAGCTCGTCCATGCCGCCGGT-3'

Supplementary Table S2. Primers used for ChIP-qPCR.

Gene	Name	Sequence
ACT2	F	5'-TGATGCACTTGTGTGTGACAA-3'
	R	5'-AAAGAGGCATCAATTCGATCA-3'
AGC2-1	A-F	5'-GCTTTAACGCTCGAAGGCCG-3'
	A-R	5'-GACAAACACGTGGTGTTCTAGAG-3'
	B-F	5'-GCGGAGCTTGTATTAGCACTTG-3'
	B-R	5'-GCGGCGTTCTTGGAGCTAGATTTG-3'
AT3G28510	A-F	5'-GGTGTGCCACGTTAATTTAGACC-3'
	A-R	5'-GTCGTCTTGTTTAGTATGCTCGG-3'
	B-F	5'-GATCCCAAAGAGCAAGCCTAG-3'
	B-R	5'-CTACATGTGTTCAGGAAACTACATG-3'
AT3G46080	A-F	5'-GGAGGAAAGCACCAAGAACATTCC-3'
	A-R	5'-CCATTTGTGACTTGCTGCGTAAGG-3'
	B-F	5'-GTGAGAAAGCCTCACCAGGCAC-3'
	B-R	5'-CGAATCTAAGTCCAAACAAGCCACTC-3'
GRXS13	A-F	5'-GGACGTGTACTGGGTAGTGGGTAC-3'
	A-R	5'-GTCTTCGTAAGGTTACGTTTTATGG-3'
	B-F	5'-GATCAAGCAACCTAGTTGTGATGG-3'
	B-R	5'-CTCAACCACCACTGGATTCACGCC-3'
IBH1	A-F	5'-GAGAGAAAGGAAAGTGGAGGTG-3'
	A-R	5'-GGAGTGAAACCAAATGAATAAGAAGG-3'
	B-F	5'-CCTCCAATCCCTCTCAAATCTCAG-3'
	B-R	5'-GCAAGAGGGCTCTGCTCCATAG-3'
INVH	A-F	5'-GCAAGCATCGTCTTTCACGG-3'
	A-R	5'-CAAGTGGTCTCCCCACGTTC-3'
	B-F	5'-GCAGAAAAGTACTGACCAGAATCAAC-3'
	B-R	5'-GTGTTGTGGTTCCAGAGTTGG-3'
LSU3	A-F	5'-CGTGTTTCATTGGTGCGACG-3'
	A-R	5'-GAATCGGTGAACGTCGTGGAG-3'
	B-F	5'-GAACGGAGAGTTGGAGAGAGAA-3'
	B-R	5'-GCCTGATCTAAAGACTCGACCT-3'
LURP1	A-F	5'-GCATGTATCTACTATCTCTCCCACCT-3'
	A-R	5'-CTTAGAGCATCTCCAGTGGTTGGT-3'
	B-F	5'-CGGAGGAGGGTGCTCTACTATAC-3'
	B-R	5'-CTTCTCCTCTACGTTGTTAGCC-3'
NHO1	A-F	5'-CTCCACCGGATTGGATGATG-3'
	A-R	5'-CCAATGAAAGAGAGCCACGTG-3'

	B-F	5'-GCATTGTCCGTGAAGCATTGG-3'
	B-R	5'-GCTATCAAGAAAGGGGATGCC-3'
NIMIN-1	A-F	5'-GTGACATCATCTCGTAACCGC-3'
	A-R	5'-AGGGACCAGGGGTAAAAGAGT-3'
	B-F	5'-CCTAGAGACCATGAGCAAGGATG-3'
	B-R	5'-CCGTGCTTCTTGATAGTGTTTG-3'
SARD1	A-F	5'-CCAATCGGGTGGGAAGATCG-3'
	A-R	5'-CTGGCAATATCCAAAGAAGTCCG-3'
	B-F	5'-CTTGCAGGCCAATTTCCAGTG-3'
	B-R	5'-CTTACAACTTTTCTAATAACGGGCTC-3'
SPL8	A-F	5'-CCCACGCCATTACCAATTACAAAA-3'
	A-R	5'-CTCACGCGCTGCTATCTCTAC-3'
	B-F	5'-CACTACCACAGAAGGCACAAAG-3'
	B-R	5'-GTACGGACGAAGAGAAGAGAAGATAG-3'
WRKY38	A-F	5'-GTTCTGACGTCAATCTGCTGAATC-3'
	A-R	5'-GCGATGTAGCTGGCGAGTGG-3'
	B-F	5'-TCACGCATATAAGTCTAGCAGAGC-3'
	B-R	5'-ACGTTCCCAAATGACTTTGC-3'
WRKY62	A-F	5'-TTATTCGCCGTTCCATCTTC-3'
	A-R	5'-TCGTCGCGTAAAATCAACTG-3'
	B-F	5'-CCAAGTCCGTCCTCCATTGTT-3'
	B-R	5'-AGCTCTCAAGCACAGGAGAAGA-3'
WRKY63(ABO3)	A-F	5'-CTCTTCATTTGTCCTCGACTGG-3'
	A-R	5'-GATTTACACACATGTTCAATGTTGAC-3'
	B-F	5'-CATTTTCGAGCTAGGGAACTTTC-3'
	B-R	5'-CAGCCTTGTGATCGATGTTTG-3'
WRKY70	A-F	5'-GGACCCTAAGTTTGGATTTCAGC-3'
	A-R	5'-TGTGTGAGGAAATGAGATGGAAC-3'
	B-F	5'-AGGAGATGGGTTCGAAGGTA-3'
	B-R	5'-TCGTTGAAGGCCATGACTTA-3'
WRKY75	A-F	5'-GCACGGATAAAATGATGACGTTCGAC-3'
	A-R	5'-GCATGCACCGACGTAGAACACAG-3'
	B-F	5'-GGAATTCAGGTGGATCGGTCTG-3'
	B-R	5'-TCCTTGTTTGAAACGCATACCTTTGTT-3'

Supplementary Table S3. Primers used for RT-qPCR.

Gene	Name	Sequence
UBQ10	qUBQ-F	5'-GGCCTTGTATAATCCCTGATGAATAAG-3'
	qUBQ-R	5'-AAAGAGATAACAGGAACGGAAACATAGT-3'
PR1	qPR1-F	5'-GCCGTGAACATGTGGGTTAG-3'
	qPR1-R	5'-GGCACATCCGAGTCTCACTG-3'
